# Impact of Dissolved Oxygen during UV-Irradiation on the Chemical Composition and Function of CHO Cell Culture Media

**DOI:** 10.1371/journal.pone.0150957

**Published:** 2016-03-14

**Authors:** Sarah M. Meunier, Biljana Todorovic, Emma V. Dare, Afroza Begum, Simon Guillemette, Andrew Wenger, Priyanka Saxena, J. Larry Campbell, Michael Sasges, Marc G. Aucoin

**Affiliations:** 1 Waterloo Institute for Nanotechnology, Department of Chemical Engineering, University of Waterloo, Waterloo, Ontario, Canada; 2 Trojan Technologies, London, Ontario, Canada; 3 SCIEX, Concord, Ontario, Canada; Centro Cardiologico Monzino, ITALY

## Abstract

Ultraviolet (UV) irradiation is advantageous as a sterilization technique in the biopharmaceutical industry since it is capable of targeting non-enveloped viruses that are typically challenging to destroy, as well as smaller viruses that can be difficult to remove via conventional separation techniques. In this work, we investigated the influence of oxygen in the media during UV irradiation and characterized the effect on chemical composition using NMR and LC-MS, as well as the ability of the irradiated media to support cell culture. Chemically defined Chinese hamster ovary cell growth media was irradiated at high fluences in a continuous-flow UV reactor. UV-irradiation caused the depletion of pyridoxamine, pyridoxine, pyruvate, riboflavin, tryptophan, and tyrosine; and accumulation of acetate, formate, kynurenine, lumichrome, and sarcosine. Pyridoxamine was the only compound to undergo complete degradation within the fluences considered; complete depletion of pyridoxamine was observed at 200 mJ/cm^2^. Although in both oxygen- and nitrogen-saturated media, the cell culture performance was affected at fluences above 200 mJ/cm^2^, there was less of an impact on cell culture performance in the nitrogen-saturated media. Based on these results, minimization of oxygen in cell culture media prior to UV treatment is recommended to minimize the negative impact on sensitive media.

## Introduction

Central to most biopharmaceutical production processes is the culture of cells in a nutrient rich solution–a solution that brings together many constituents to satisfy the growth and production requirements of the cells. The production of such a solution results in a complex mixture that could have entrained adventitious agents from some or any of its constituents. While filtration is commonly applied to guard against contamination, there are small organisms that cannot be easily removed by filtration. Minute mouse virus (~20 nm), mycoplasma (~100 nm), and leptospira (~100 nm) are all organisms that are too small to be filtered from media and have been detected in cell culture [1–3].

Viral contamination has been detected in drug products, and could have serious ramifications for the patients and the producer. Viral contamination of a rotavirus vaccine (Rotarix^®^, GSK) by a porcine circovirus, a yellow fever vaccine by avian retrovirus type C, and a poliovirus vaccine by simian vacuolating virus (SV40), are examples that have caused adventitious agents to become a major concern for the biopharmaceutical industry [4,5]. These types of contamination compromise patient safety; cause the shutdown of production facilities; result in drug shortages; weaken public perception of pharmaceuticals; lead to legal ramifications and, ultimately, significant financial losses; as well as usher new stricter regulations [4]. To mitigate contamination concerns for biopharmaceutical production, additional barriers to adventitious agents are critical [4].

Ultraviolet (UV) irradiation is known to be an effective disinfection technique for waterborne pathogens, such as viruses and bacteria [6]. However, cell culture media has unique optical properties and constituents which must be considered since UV treatment can induce significant chemical reactions via production of reactive oxygen species. In a recent study, we showed that using a collimated beam to UV-irradiate chemically defined media exposed to atmosphere resulted in little to no impact on the ability to culture Chinese hamster ovary (CHO) cells compared to media exposed only to visible light [7]. Though powerful in its proof-of-principle, the implementation of such a system in a biopharmaceutical setting is near impossible. Typical UV irradiation research studies utilize batch reactors (i.e., collimated beam devices) [7–10]; however, continuous-flow reactors are significantly more desirable for industrial processes.

Through this study, using a novel continuous flow UV reactor, the effect of dissolved oxygen during UV irradiation of a chemically defined media on the chemical composition and functionality of the media is investigated.

## Materials and Methods

### Experimental Design

UV irradiation of commercial serum-free, chemically defined CHO growth media (CD-CHO, Life Technologies Inc., ON, CA) supplemented with Gibco^®^ GlutaMAX^™^ and HT supplement, was achieved using two identical continuous-flow UV reactors, designed by Trojan Technologies (ON, Canada), connected in series. A peristaltic pump was used to move fluid through Teflon tubing arranged around the central UV lamps of the reactors. Control samples used throughout this study refer to media that was passed through the reactor system with the UV lamp turned off, and were therefore treated with a fluence of 0 mJ/cm^2^. Three fluences (UV doses) were considered: 200 mJ/cm^2^, 400 mJ/cm^2^, and 600 mJ/cm^2^. Increasing fluences were achieved by flowing media through the UV reactors in series for one, two, or three passes. The fluence was quantified via the log reduction of MS2, the bacteriophage used as the challenge organism in this study. To evaluate the impact of oxygen saturation on the effects of UV irradiation, the media was irradiated under oxygen- or nitrogen-saturated conditions.

### UV Irradiation

**Fig 1:**
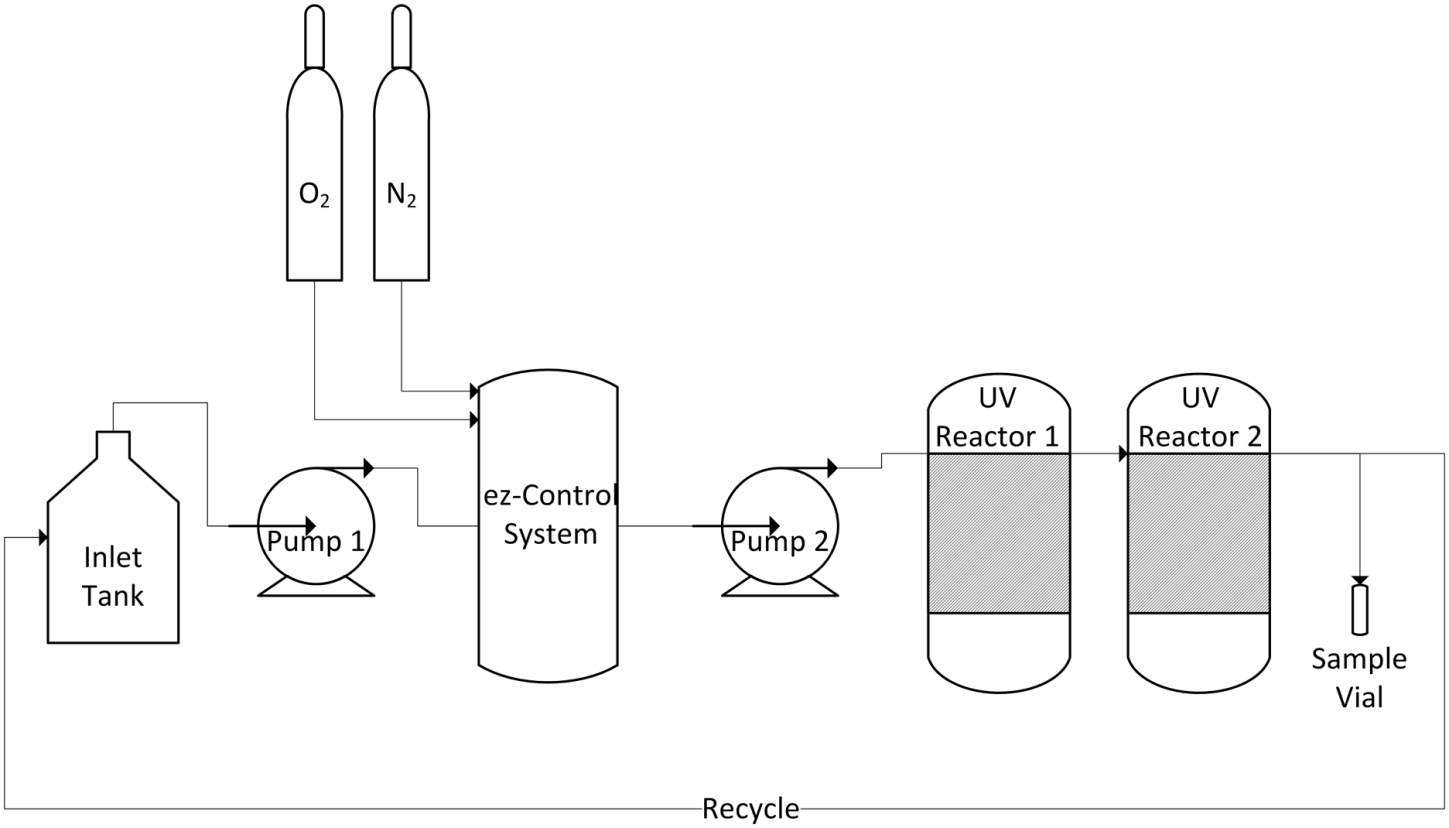
Experimental apparatus.

The experimental system was designed to allow control of the oxygen saturation prior to UV irradiation (Fig 1). An ez-Control system (Applikon Biotechnology B.V., Delft, NL) was used to control the oxygen saturation level in the media as well as monitor the media pH and temperature. Using the ez-Control system, the media was prepared at two oxygen levels–oxygen-saturated and nitrogen-saturated.

To achieve oxygen-saturation, high-purity compressed O_2_ gas flowed from a regulated compressed gas cylinder (Praxair Canada, Inc., Mississauga, ON) through the ez-Control system at 20 mL/min into the bioreactor until the dissolved oxygen reached a steady maximum equilibrium. For nitrogen saturation, high-purity compressed N_2_ gas flowed from a regulated compressed gas cylinder (Praxair Canada, Inc., Mississauga, ON) with 100% aeration through the ez-Control system with rotameter control at approximately 20 mL/min flow rate into the bioreactor until the dissolved oxygen reached a steady minimum equilibrium.

Upon equilibration at the desired oxygen level, the media was irradiated using a continuous-flow reactor with fluid pumped around a central low-pressure mercury UV lamp emitting at 254 nm. The reactor system was designed to achieve good mixing and uniform fluence to the fluid. To achieve the desired fluence, the media was passed through the two-reactor system at 50 mL/min. After discarding a volume of fluid equal to three UV system volumes, irradiated media was collected for analysis or collected to be pumped back to the ez-Control system and re-equilibrated with the appropriate gas so that it could be passed through the UV reactor system multiple times. The system was operated in batch mode, rather than recirculation, to ensure accurate dose delivery. One pass through two UV reactors delivered a fluence of approximately 200 mJ/cm^2^, two passes delivered 400 mJ/cm^2^, and three passes delivered 600 mJ/cm^2^. The actual fluence delivered was verified using the procedure described in the UV Fluence section.

### UV Fluence

The fluence, quantified as reduction equivalent fluence (REF) or dose (RED) [11], delivered to the cell culture media was determined using a viral clearance test with the challenge organism, MS2, inoculated in the culture media. MS2 is a well characterized bacteriophage and is used extensively to validate UV disinfection systems for drinking water [12]. The fluence was quantified using a similar experimental set-up, but with only one reactor–the media was saturated with the appropriate gas and passed through one UV reactor at two flow rates (50 mL/min and 100 mL/min). The oxygen-saturated and nitrogen-saturated results were compared to ensure that there was no change in the measured fluence based on the saturation gas. The log reduction in MS2, which is used to calculate the fluence delivered by the reactor, was determined by GAP EnviroMicrobial Services (ON, Canada), who also provided the bacteriophage. The single reactor fluence was then multiplied by the number of reactor passes executed (i.e., 2, 4, or 6). This approach assumes that the UV doses are additive, which is a good approximation for well-mixed reactors such as the one used in this study.

### NMR Spectroscopy and Metabolite Profiling

To prepare samples for NMR analysis, 70 μL of internal standard (99.9% D_2_O with 5 mM 4,4-dimethyl-4-silapentane-1-sulfonic acid) and 0.2% w/v sodium azide were added to 630 μL of media. The prepared samples were mixed in a vortex mixer and pipetted into 5 mm NMR tubes (NE-UL5-7, New Era Enterprises Inc., NJ, US). The NMR spectra were acquired with a 600 MHz Avance spectrometer equipped with a TXI 600 triple resonance probe (Bruker, MA, US) using the first increment of the NOESY pulse sequence with a 1 s pre-saturation pulse, followed by a 4 s acquisition time. The Chenomx NMR Suite 7.7 (Chenomx Inc., AB, Canada) was used to process the spectra, and baseline, phase, shim, and chemical shape corrections were performed manually using the software. The compounds were identified and quantified by targeted profiling with the built-in library of chemical resonances and the internal standard as a reference compound. Additional information regarding the profiling of cell culture media can be found elsewhere [13].

### LC-MS Analysis

#### Standards and Sample Preparation

Analytical grade vitamin standards and Supelco Supelclean LC18 (500 mg / 3 mL) SPE cartridges were purchased from Sigma Aldrich (Oakville, ON). Optima LC-MS grade water and acetonitrile, and HPLC grade methanol, were purchased from Fisher Scientific (Canada). Phenex RC 0.2 μm syringe filters were purchased from Phenomenex (CA, US).

Calibration curves were developed for each vitamin using serial dilutions of standards, between 0.025 and 250 ppb. Stock solutions were prepared weekly by dissolving in water or ammonium hydroxide, as appropriate, and stored at 4°C. Working solutions were prepared daily, and vortexed prior to use.

Media samples were diluted 10:1 with HPLC grade water, and a solid-phase-extraction (SPE) method was used to remove compounds that would otherwise interfere with the quantification of vitamins. Supelco Supelclean LC18 SPE cartridges were used to extract the vitamins. Cartridges were conditioned prior to use with 3 mL of methanol and 3 mL of water at a flow rate of 3 mL/min. After extraction, 3 mL of 85% methanol was used as an elution solvent. The methanol was subsequently evaporated using a rotary evaporator, and then reconstituted with water to a final dilution of 100x. Finally, samples were filtered with a Phenex 0.2 μm syringe filter prior to analysis.

#### Liquid Chromatography

The LC-MS system comprised an Agilent 1100 HPLC (Santa Clara, CA, US) equipped with an autosampler and thermostated sample chamber, utilizing an Agilent Poroshell 120, 2.7 μm SB-C18, 3.0 × 50 mm analytical column. An optimized gradient elution method was used to achieve good chromatographic separation. The gradient was formed utilizing two solvents. Solvent A consisted of a 5 mM ammonium formate in water with 0.1% formic acid. Solvent B consisted of 5 mM ammonium formate in 95% acetonitrile in water with 0.1% formic acid. The chromatographic run was carried out over 14 min with 100% A up to 5 min, then 25% of B at 5.10 min, 43% of B at 6.60 min, followed by 98% B from 6.70 to 8 min, and back to 100% A from 8.10 to 14 min.

#### Mass Spectrometry

The mass spectrometer was a SCIEX 6500 triple quadrupole mass analyzer (Concord, ON). Multiple Reaction Monitoring (MRM) was used as the mass spectrometer’s detection mode, with unique ion precursor and fragment ions monitored for each vitamin; these MRM transitions and their optimized collision energies (CEs), declustering potentials (DPs), and entrance potentials (EPs) are listed in S1 Table. Other mass spectrometer parameters, including ion source conditions, are listed in S2 Table. Statistical data analysis was conducted using Analyst^®^ 1.6.2 software (SCIEX, ON, Canada) using the following parameters for processing calibration curve data: Gaussian smooth width– 3 points, noise percentage– 50%, peak splitting factor– 2 points, and a weighting of 1/x^2^.

### Cell Culturing

Cell culture was performed with CHO^BRI^ cells (Biotechnology Research Institute, QC, CA) adapted to grow in a commercial serum-free, CD-CHO growth media (Life Technologies Inc., ON, CA) supplemented with Gibco^®^ GlutaMAX^™^ (10 mL per 1 L CD-CHO), HT supplement (10 mL per 1 L CD-CHO), Pluronic F-68 non-ionic surfactant (10 mL per 1 L CD-CHO), and anti-clumping agent (10 mL per 1 L CD-CHO) (Life Technologies, Inc., ON, Canada). Both GlutaMAX^™^ and HT supplement were added to the media prior to UV irradiation, while Pluronic F-68 and anti-clump were added prior to inoculation (after UV irradiation). The parental culture was incubated at 37°C and 5% CO_2_ with agitation at 120 rpm. Erlenmeyer flasks (125 mL non-pyrogenic polycarbonate, Corning Inc., NY, US) with a vented cap were used for the parental and experimental cultures, and the working volume of the flasks was 30 mL. When the mother flask reached a viable cell density of 2 to 3 × 10^6^ cells/mL, the experimental cultures were inoculated with a seeding density of 0.2 × 10^6^ viable cells/mL. Experimental cultures for each treatment group were inoculated and cultured in triplicate. Cell density and viability were monitored daily for up to eight days using a haemocytometer by trypan blue exclusion. When the cells were no longer viable, the cell pellet and supernatant were separated by centrifugation at 500 g for 10 min.

### Statistical Analysis

For all data collected, a two-tailed Student’s t test with 95% confidence interval was used for statistical analysis, except for NMR results where regression analysis was used to determine compounds showing significant trends with increasing UV fluence. For those compounds determined to have significant regression, further analysis of slope comparison between oxygen-saturated and nitrogen-saturated samples was conducted. Regressions were made using the R programming language. For LC-MS/MS data, the statistical analysis was performed using Analyst^®^ 1.6.2 software (SCIEX, ON, Canada).

## Results and Discussion

Based on published results [7], it was expected that the high UV doses applied in this study could have impacts on the properties and performance of the cell culture media. The irradiated media was evaluated for changes in chemistry, and the cell culture performance in the media was also assessed.

### UV Fluence Quantification

Since UV inactivation kinetics are often first order, they can be characterized by a single parameter. UV sensitivity of bacteria and viruses is often characterized by the D_10_ value–the UV fluence required to reduce the microorganism population by one log. For example, MS2, a non-enveloped bacteriophage often used to evaluate the potential for virus inactivation via UV irradiation [14], requires a fluence of approximately 23 mJ/cm^2^ for one log reduction of the population [8]. The UV sensitivity of many adventitious agents has been studied and quantified. The common bacterial contaminant, mycoplasma, is relatively sensitive to UV, with a D_10_ of about 1 mJ/cm^2^ [15]. MMV, a common virus in laboratory mice that can replicate in CHO cell lines [16], has a D_10_ value of approximately 2.2 mJ/cm^2^ [10]. By contrast, adenovirus type 40, a cause of gastroenteritis in children, is much more resistant to UV irradiation with a D_10_ value of approximately 55 mJ/cm^2^ [9].

**Fig 2:**
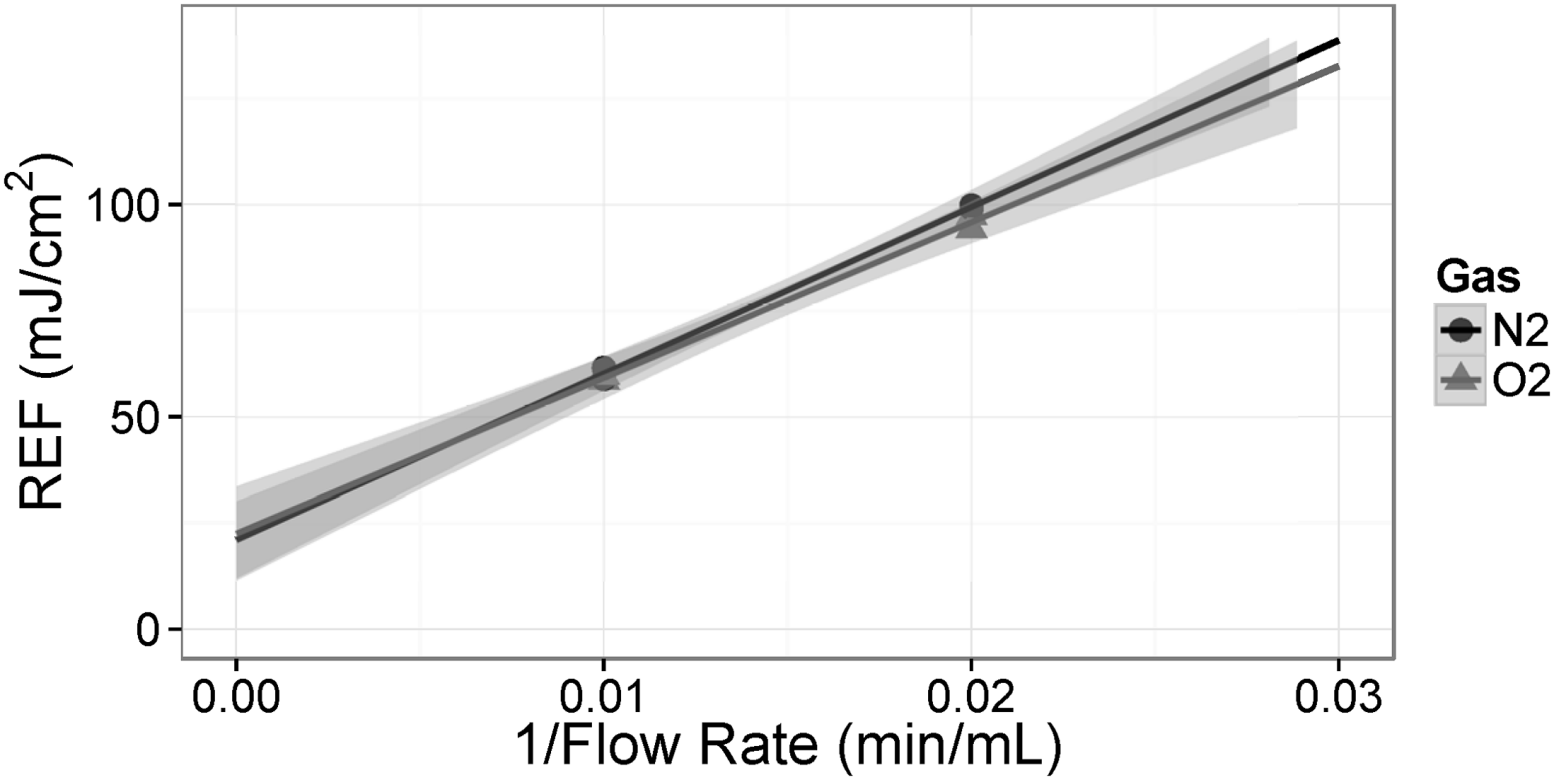
Reduction equivalent fluence (REF) based on MS2 bioassay of irradiated CD-CHO media with one UV reactor at two flow rates (50 mL/min and 100 mL/min) for both oxygen- and nitrogen-saturated media.

The double reactor set-up in this work was used to apply very large fluences to the media to test the limits of UV irradiation. The very high doses used in this study preclude the direct use of MS2 inactivation to quantify the delivered fluence, since the maximum achievable titre of the starting solution (approximately 10^9^ /mL) would be completely eliminated. Therefore, the multi-pass fluence is estimated based on an extrapolation of a single-reactor, single-pass analysis of the surviving MS2 quantities. No difference was observed between the surviving MS2 quantities from the irradiated oxygen- and nitrogen-saturated media (Fig 2). The single reactor 50 mL/min REF was 97.7 mJ/cm^2^. Assuming additive properties, the fluence values for the double reactor from one, two, and three passes are estimated to be 195, 390, and 585 mJ/cm^2^, respectively (Table 1).

**Table 1:** Fluence rates estimated based on the single reactor pass clearance of MS2 at 50 mL/min.

Description	Number of Reactor Passes	Reduction Equivalent Fluence (mJ/cm^2^)
**Single reactor**	1	97.7
**Double reactor single pass**	2	195
**Double reactor double pass**	4	390
**Double reactor triple pass**	6	585

### Optical Properties

The absorption and scattering coefficients at 254 nm were determined based on transmittance and reflectance measurements from a Cary 300 spectrophotometer with a 6″ integrating sphere (Agilent Technologies, CA, US) to include scattered light. Samples were collected for analysis from each of the fluences (control, 195 mJ/cm^2^, 390 mJ/cm^2^, and 585 mJ/cm^2^) for both the oxygen- and nitrogen-saturated media. There were no significant differences between the absorption and scattering coefficients for any of the samples, indicating that the UV treatment does not affect the optical properties of the cell culture media at 254 nm.

### Metabolic Profiling

Upon exposure to light, riboflavin and other photosensitizers can increase the levels of reactive oxygen species and lead to damage of cell culture media [17]. With oxygen present in cell culture media, the riboflavin or reactive oxygen species could participate in photooxidation of amino acids (e.g., tryptophan), and lead to decreased cell growth [18]. Therefore, understanding the repercussions of UV irradiation for cell culture media in the presence and absence of oxygen is of utmost importance for industrial use of UV disinfection techniques. Studies suggest that there may be negative effects of UV irradiation of cell culture media, including changes in the substrate concentrations and the formation of by-products, which could affect the properties of the final product [19,20].

**Fig 3:**
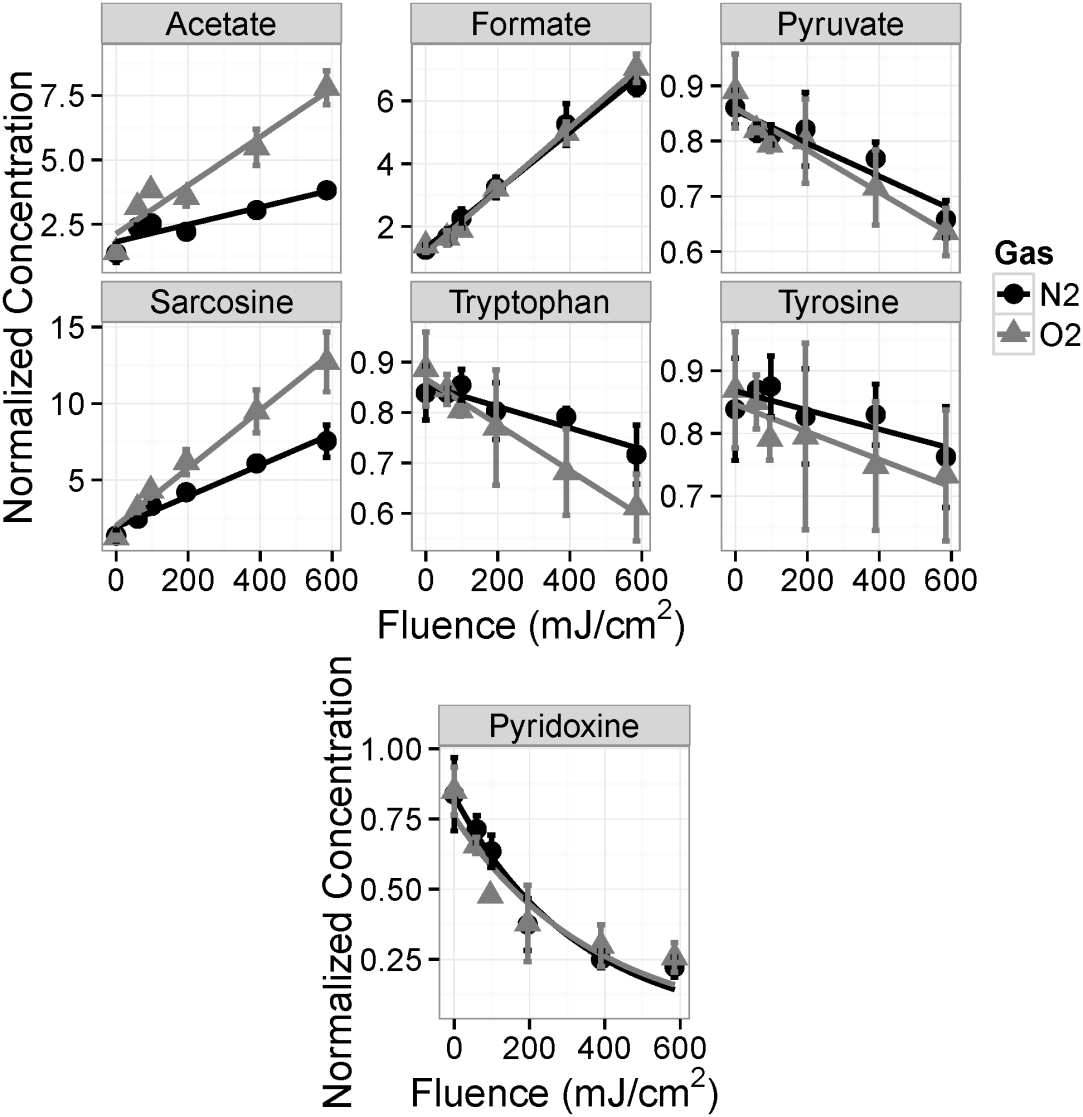
Concentration change with respect to UV fluence for oxygen-saturated (light gray triangles) and nitrogen-saturated (dark gray circles) media for compounds with statistically significant regression trends. The error bars represent the standard deviation where N = 10.

Of the 30 compounds in the media identified and quantified by NMR, seven compounds consistently had statistically significant trends with respect to UV irradiation: acetate, formate, pyridoxine, pyruvate, sarcosine, tryptophan, and tyrosine (Table 2 and Fig 3). For these compounds, except pyridoxine, the concentration changes are linearly proportional to the applied UV fluence; for pyridoxine, a low-concentration plateau is observed at high fluences.

**Table 2:** Percent concentration change per 100 mJ/cm^2^ of UV fluence for both oxygen- and nitrogen-saturated media for compounds with statistically significant trends. Linear regression was used to determine the concentration change per 100 mJ/cm^2^ REF. The confidence intervals respresent the standard deviation of N = 10 measurements.

Compound	Concentration change per 100 mJ/cm^2^ under oxygen-saturation (%)	Concentration change per 100 mJ/cm^2^ under nitrogen-saturation (%)
**Acetate**	76.5 ± 2.2	39.3 ± 1.9
**Formate**	62.0 ± 1.5	77.7 ± 2.3
**Pyridoxine**	Non-linear	Non-linear
**Pyruvate**	-5.1 ± 0.2	-3.3 ± 0.3
**Sarcosine**	155.5 ± 4.4	92.5 ± 7.0
**Tryptophan**	-5.7 ± 0.3	-1.1 ± 0.4
**Tyrosine**	-2.9 ± 0.4	-0.2 ± 0.8

Irradiation under low oxygen conditions resulted in smaller changes in composition. For each of these compounds, the absolute concentration change with fluence is either the same between the nitrogen- and oxygen-saturated samples (formate and pyridoxine) or less for the nitrogen saturated samples (acetate, pyruvate, sarcosine, tryptophan, and tyrosine) (Fig 3). This indicates that the media is more resistant to UV damage under nitrogen saturation than when exposed to oxygen. It is speculated that higher concentrations of reactive oxygen species (ROS) are generated by UV irradiation in the oxygen-saturated media, and that these ROS are responsible for the greater changes in concentration.

The concentrations of pyridoxine, pyruvate, tryptophan, and tyrosine decrease as the fluence increases, indicating the destruction of these compounds with UV exposure (Fig 3). Conversely, acetate, formate, and sarcosine are present in increasing amounts with increasing UV fluence, indicating that these compounds are produced from the UV-initiated photoreactions (Fig 3). The three compounds with the largest relative concentration changes are acetate, pyruvate, and sarcosine, and these compounds are all produced in quantities of 40 to 155% of the original concentration per 100 mJ/cm^2^ of UV fluence (Table 2).

### LC-MS Analysis

**Fig 4:**
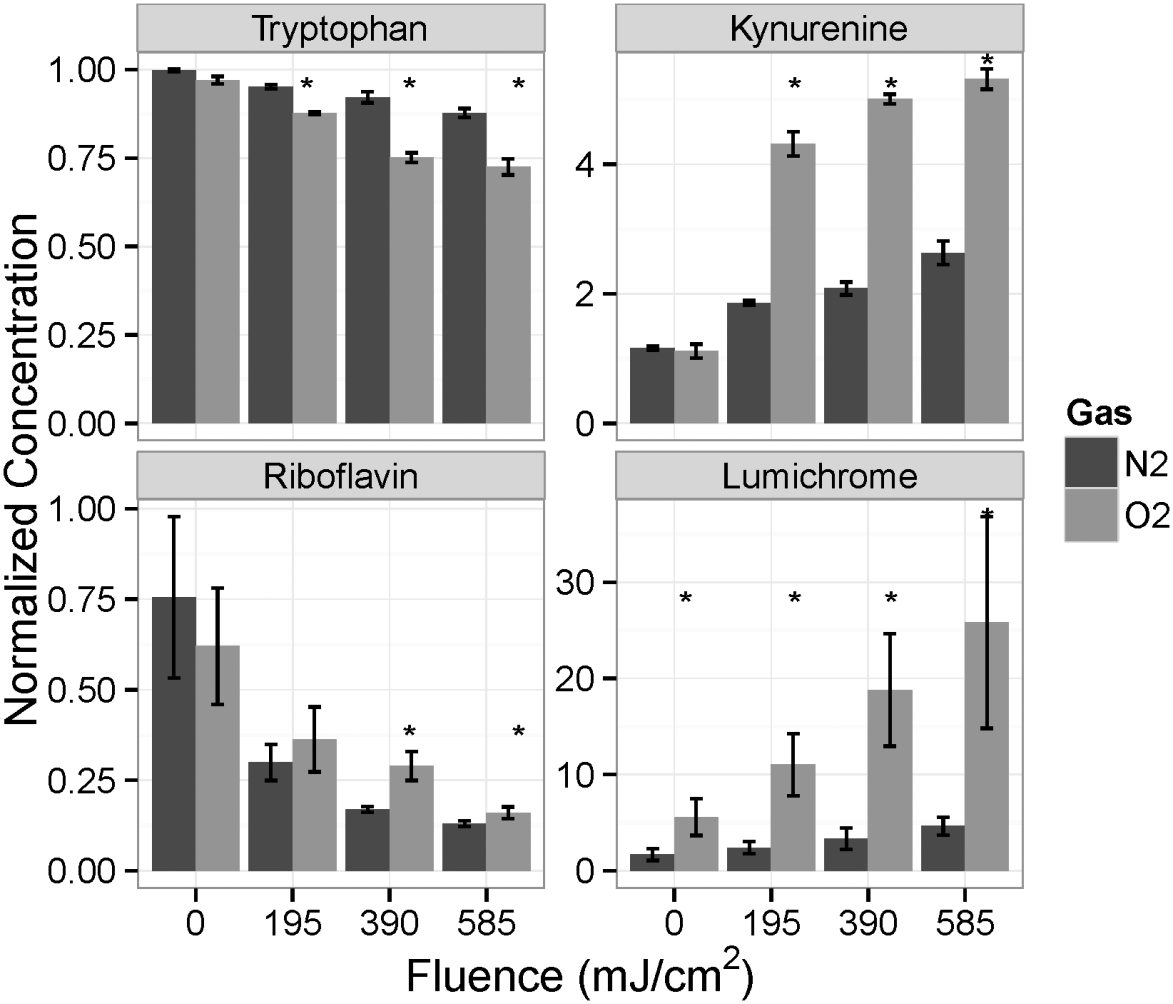
Concentration change of tryptophan, kynurenine, riboflavin, and lumichrome with respect to UV fluence for oxygen-saturated (O2) and nitrogen-saturated (N2) media. *—Significant difference between N_2_ and O_2_ saturated groups at an individual fluence level.

Concentrations of vitamins were measured by LC-MS. There were many significant differences between the nitrogen- and oxygen-saturated samples. The amount of lumichrome in the oxygen-saturated samples was significantly greater than that in the nitrogen-saturated samples (Fig 4) at all UV fluences. Under nitrogen saturation, the lumichrome concentration was nearly unaffected by UV irradiation. Lumichrome is one of the major photoproducts of riboflavin, along with lumiflavin, and its concentration might be expected to increase with the photo-degradation of riboflavin [21]. However, it can be seen that the photo-degradation of riboflavin was actually greater in the nitrogen-saturated media (Fig 4). Riboflavin is the only compound for which we observed higher degradation under nitrogen-saturated conditions. The different conversion rates for lumichrome formation may suggest that different pathways are involved in flavin degradation for the two conditions. The pH of the two solutions before irradiation was the same, so pH was not responsible for the different rates of riboflavin degradation. The rate of generation of ROS in cell culture media by light exposure is greater under conditions that allow oxygen access [22]. It is likely that, in the absence of oxygen, ROS will be less abundant leading to the reduced generation of lumichrome by intramolecular photochemistry of riboflavin. However, certain riboflavin derivatives (5-deaza-riboflavin and iso-6,7-riboflavin) have been shown to have more efficient photodegradation in anaerobic environments [23]. It is possible that in the N_2_-saturated condition, riboflavin degradation was accelerated leading to the formation of larger quantities of photoproducts other than lumichrome.

Another measured difference between the media treated under the different saturation conditions is the generation of kynurenine [24]. This compound is generated during the photolysis of tryptophan [25]. The results show that significantly more kynurenine was generated under oxygen-saturated conditions than under nitrogen saturation (Fig 4) at all UV fluences above zero. This is consistent with the greater destruction of tryptophan under oxygen saturation (Fig 4), which shows significant decreases compared to nitrogen saturated samples at all UV fluences above zero. A mechanism for this may be the ability of oxygen to accept the electron created when photoexcited tryptophan forms the tryptophan cation and the electron that can subsequently react with oxygen to form the superoxide anion. Reaction of the tryptophan cation and superoxide anion leads to N-formylkynurenine, which is unstable, and leads to kynurenine and formic acid [24].

**Fig 5:**
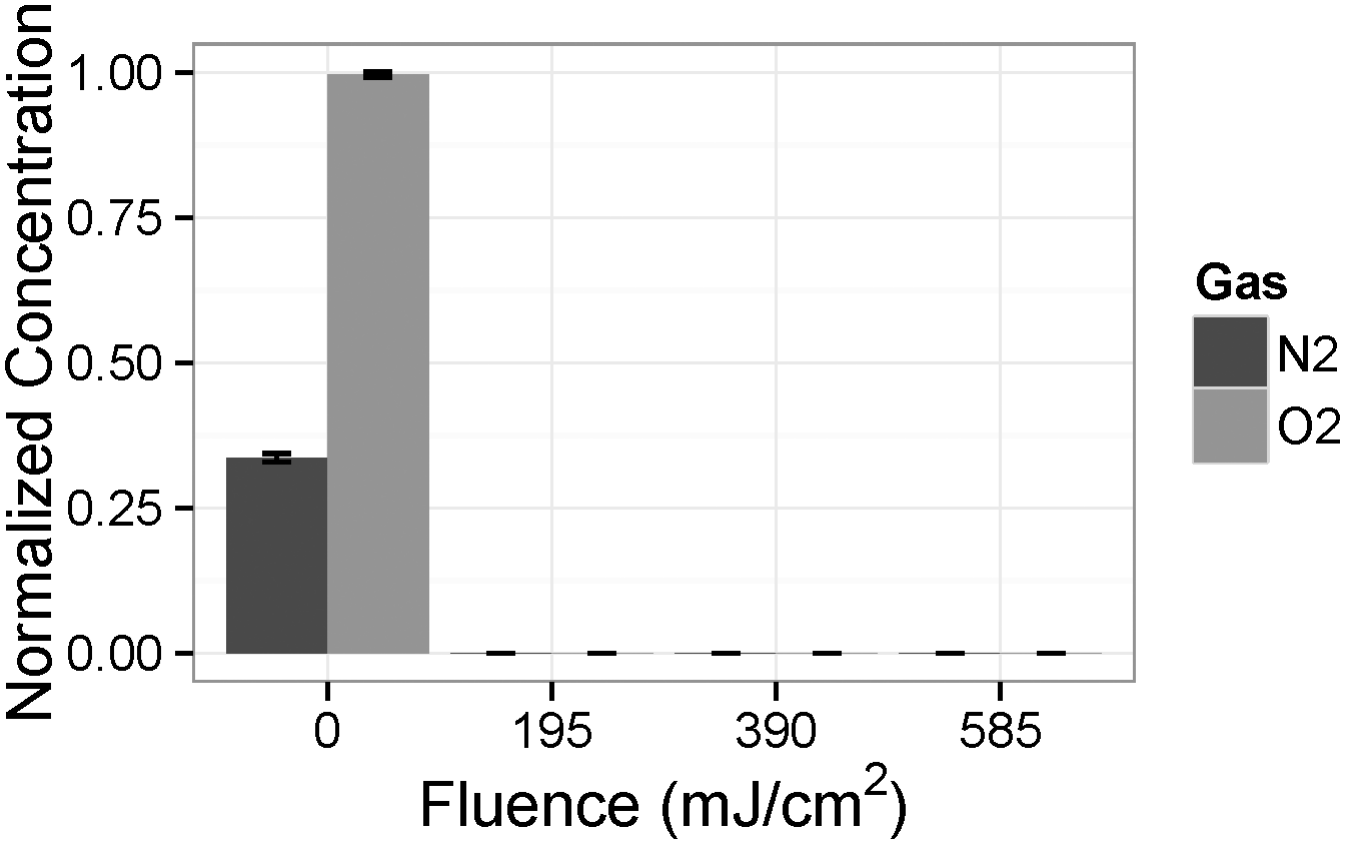
Concentration change of pyridoxamine with respect to UV fluence for oxygen-saturated (O2) and nitrogen-saturated (N2) media.

Pyridoxamine was rapidly depleted by UV irradiation of media, regardless of the oxygen saturation (Fig 5). Surprisingly, pyridoxamine levels were significantly greater in the oxygen-saturated control than the nitrogen-saturated control. We have yet to find an explanation for this; nonetheless, vitamin B6 (pyridoxine) and its derivatives are singlet oxygen quenchers and degrade in the process of quenching reactive oxygen species photo-generated from riboflavin in solution [26]. The relatively greater depletion of pyridoxamine compared with pyridoxine in the present work disagrees with the results reported showing that laboratory light caused similar amounts of depletion of pyridoxine, pyridoxal, and pyridoxamine [27]. This may be related to matrix effects, since riboflavin and tryptophan were not included in their fluid [27].

### Cell Culture Analysis

**Fig 6:**
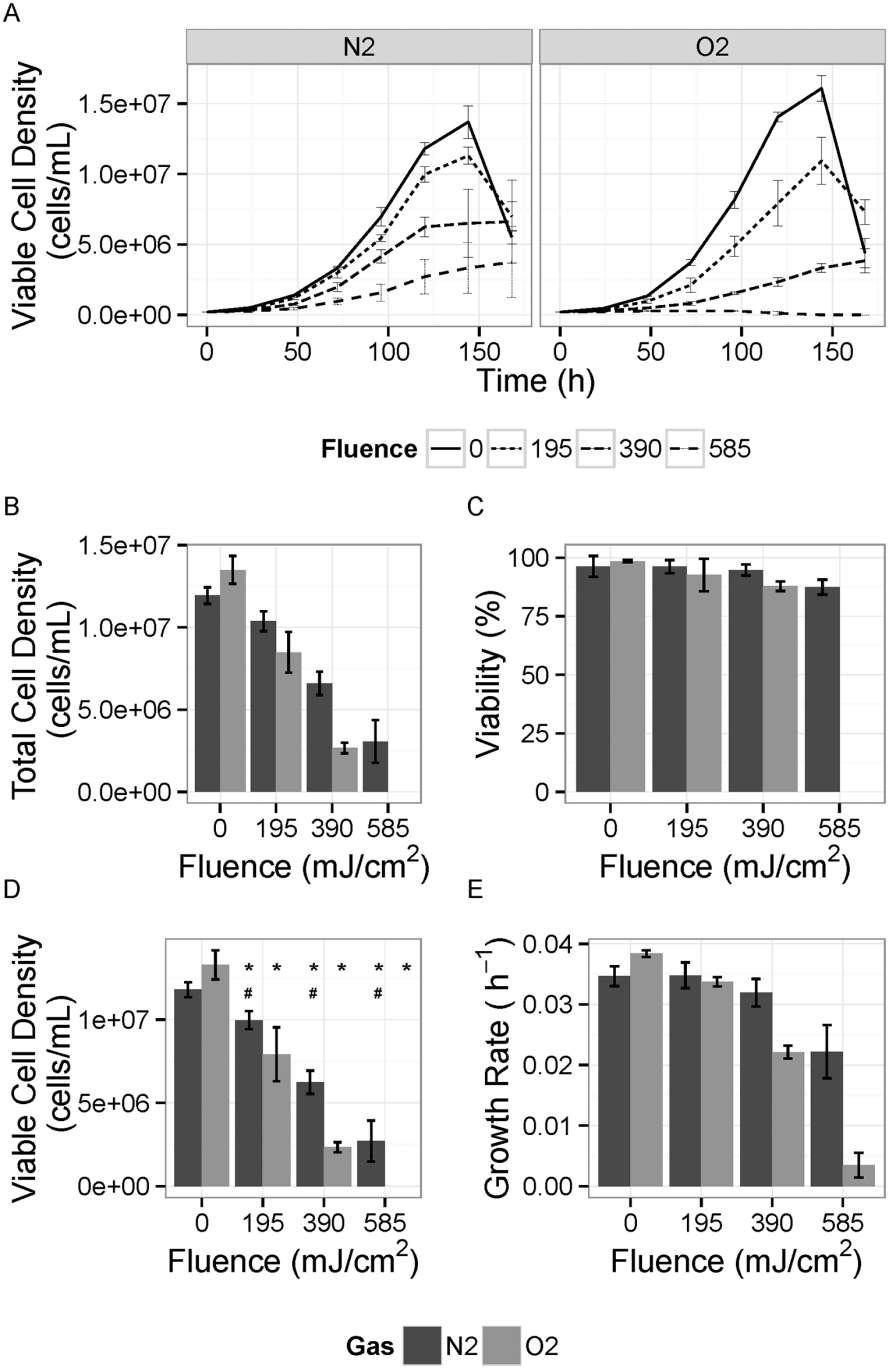
Comparison of the cell culture performance. A) Time-course of viable cell density for cells grown in different fluence UV-treated nitrogen-saturated (N2) or oxygen-saturated media (O2) media. B) Total cell densities reached on Day 5 (end of exponential phase for most cultures). C) Viability of cells on Day 5. D) Total viable cell density. E) Growth rate during exponential growth phase (Days 1 through 5). The error bars represent the standard deviation for N = 6. *—Significant difference when compared to control for the same gas treatment group. #—Significant difference between N_2_- and O_2_-saturated groups at an individual fluence level.

The performance of the irradiated cell culture media was evaluated based on the growth statistics (overall cell growth rate, total number of cells, viable cell density, and cell viability) at Day 5 (120 h) as well as the overall growth profile since the authors found that this time period provided a good indication of the cell performance in the irradiated media and represented the day when most cultures ended their exponential growth phases (Fig 6).

With increasing fluence, there was a significant increasing negative impact of UV treatment that was greater in the oxygen-saturated media. As expected based on the chemical changes in the irradiated media (Table 2 and Figs 3–5), the cell growth was affected more for oxygen-rich media at higher fluences. Under both oxygen- and nitrogen-saturation, the effect of UV treatment on cell growth was significantly different at 195, 390 and 585 mJ/cm^2^. However, other than the control, the viable cell density in nitrogen-saturated samples was significantly greater than in oxygen-saturated samples at all three UV fluences. This indicates that the media is more resistant to UV under nitrogen-saturation than oxygen-saturation.

The fluence values applied in this study are relatively high, sufficient to provide a significant level of viral clearance. Even the lowest applied fluence of 195 mJ/cm^2^ could achieve 3.5 log reduction of adenovirus type 40 (i.e., surviving fraction 3 × 10^−4^), which is relatively resistant to UV disinfection. This same dose would theoretically provide more than 10 log inactivation of MMV, leptospira, or mycoplasma. Based on these results, UV irradiation in an oxygen-void environment has the potential to minimize the chemical changes occurring in the media and allow for higher fluences providing a significant level of inactivation without significantly affecting cell performance.

## Conclusions

UV irradiation in oxygen-saturated media resulted in greater generation of kynurenine and lumichrome, consistent with the generation of more reactive oxygen species by UV irradiation under oxygen saturation. The oxygen saturation level has clearly influenced the photochemistry resulting from UV excitation of chromophores in the media. Further work will be required to determine the precise chemistry responsible for poor culture performance in the oxygen-saturated media.

For the chemically-defined media used in this study (CD-CHO), cell culture performance after UV irradiation deteriorated even at a fluence of 195 mJ/cm^2^. Depleting oxygen before irradiation results in smaller changes in the chemical composition of the media, and reduces the impact on the cell culture performance. Therefore, eliminating oxygen from cell culture media prior to irradiation represents a promising approach to allow higher levels of UV disinfection without affecting the media performance. While the results require confirmation in other types of media, including those with animal-derived components, it is likely that this method will enable UV to be applied at higher fluences for alternative media types. To critically assess the suitability of UV disinfection for cell culture media, oxygen saturation levels should be controlled and minimized prior to irradiation.

## Supporting Information

S1 Fig MRM Chromatogram for 14 vitamins at 250ug/L. (DOC)

S2 Fig Calibration Curves for vitamins (from 0.025 to 250ug/L range). (DOC)

S3 Fig CD-CHO NMR profile. Complete profile for CD-CHO media generated by NMR and analyzed with Chenomx NMR Suite 8.0.
(DOC)

S4 Fig Tyrosine NMR profile. Representative profiles of the main peaks used for estimating quatification of tyrosine in O_2_ saturated control (left panel) and 195 mJ/cm^2^ irradiated (right panel) CD-CHO.
(DOC)

S1 Table Experimental parameters. Optimized MRM transitions and related instrumental parameters for the analytes monitored in this study.
(DOC)

S2 Table Mass spectrometer parameters. Additional instrumental parameters employed with the 6500 triple quadrupole mass spectrometer.
(DOC)

S3 Table Retention times for vitamins measured by LCMS. (DOC)
