# Supplementary figures and images for: Impact of Dissolved Oxygen during UV-Irradiation on the Chemical Composition and Function of CHO Cell Culture Media
Source: PLoS One. 2016 Mar 14;11(3):e0150957. doi: 10.1371/journal.pone.0150957 (PMC4790850; doi:10.1371/journal.pone.0150957)

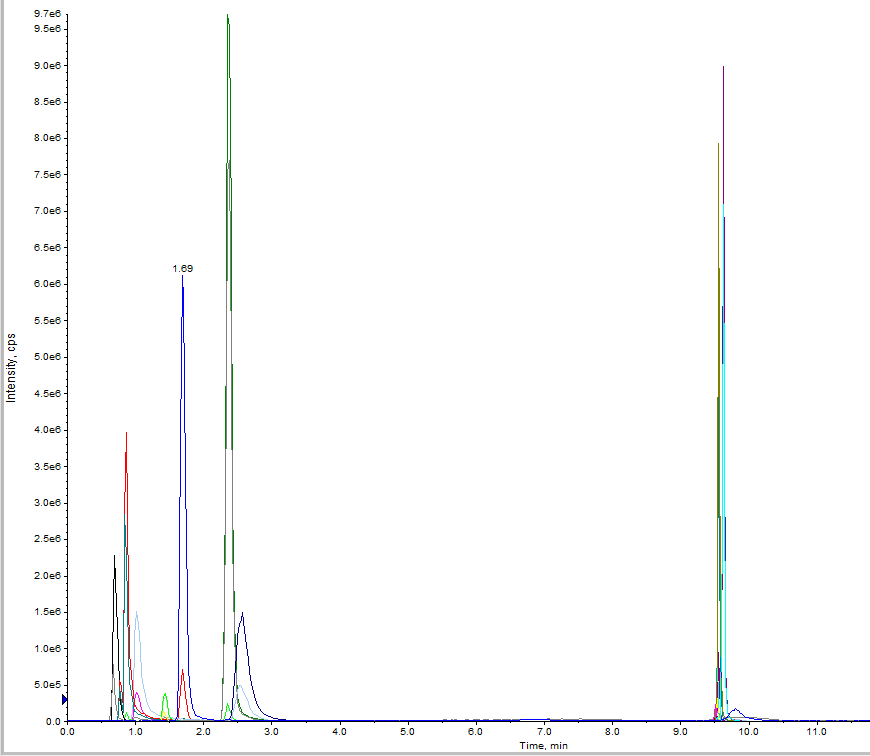

Supplement: S1 Fig — (DOC) [file pone.0150957.s001.doc]

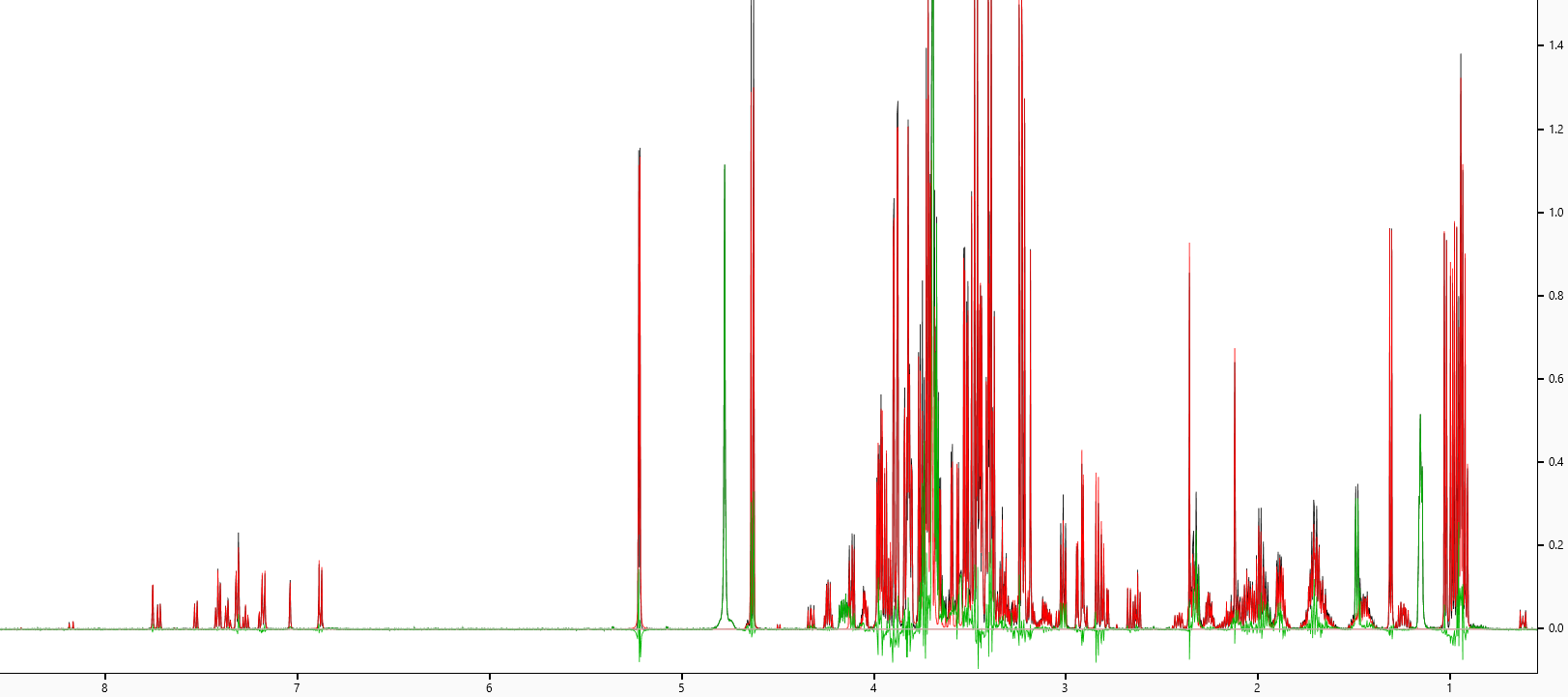

Supplement: S3 Fig — Complete profile for CD-CHO media generated by NMR and analyzed with Chenomx NMR Suite 8.0. (DOC) [file pone.0150957.s003.doc]

**O2 Saturation – Control O2 Saturation – 195 mJ/cm2**


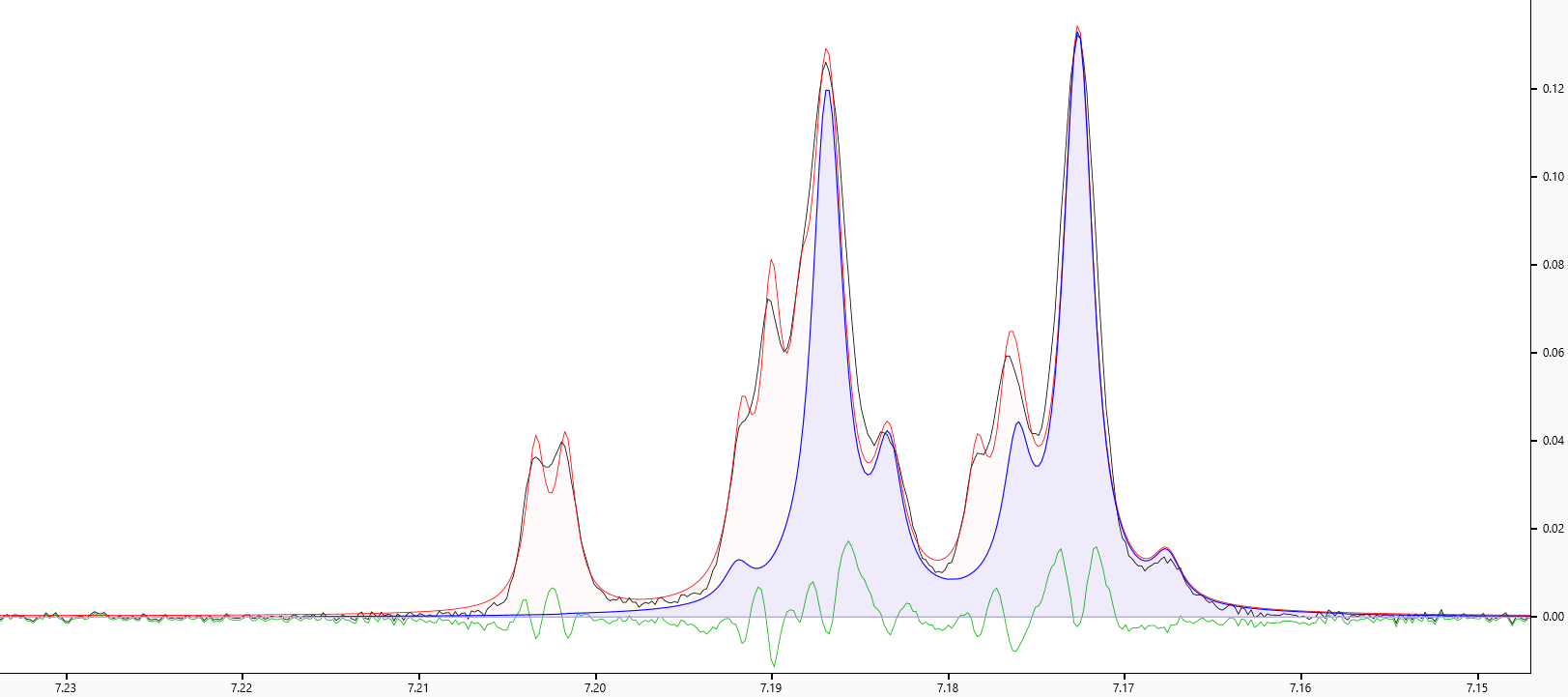

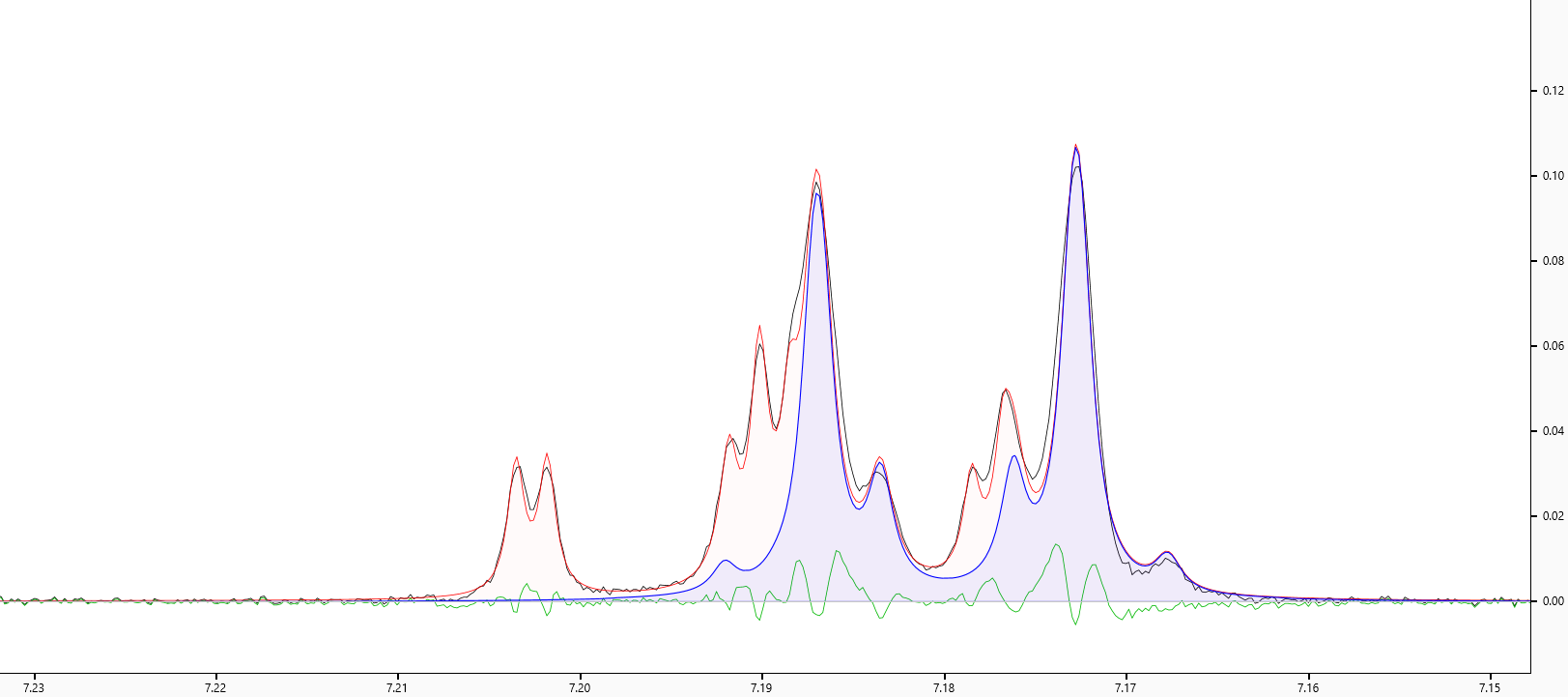


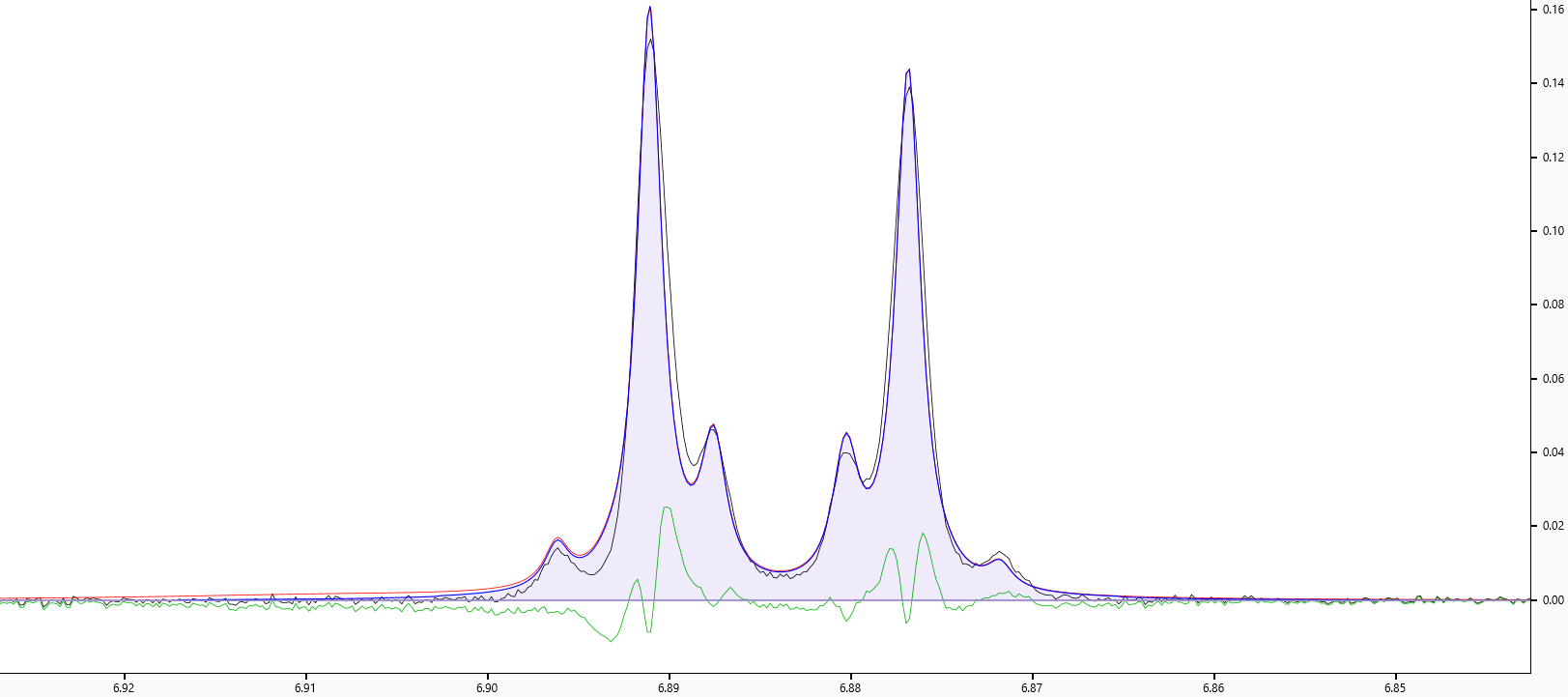

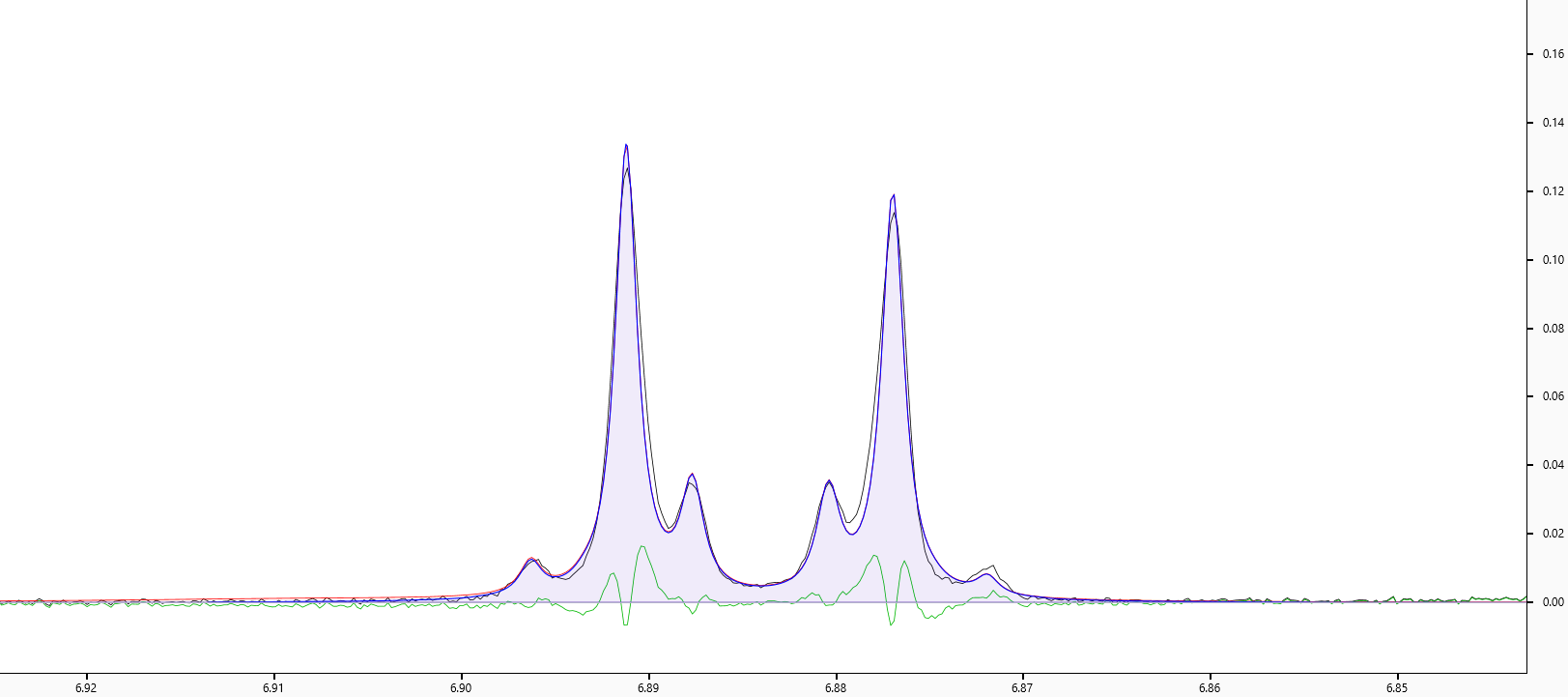


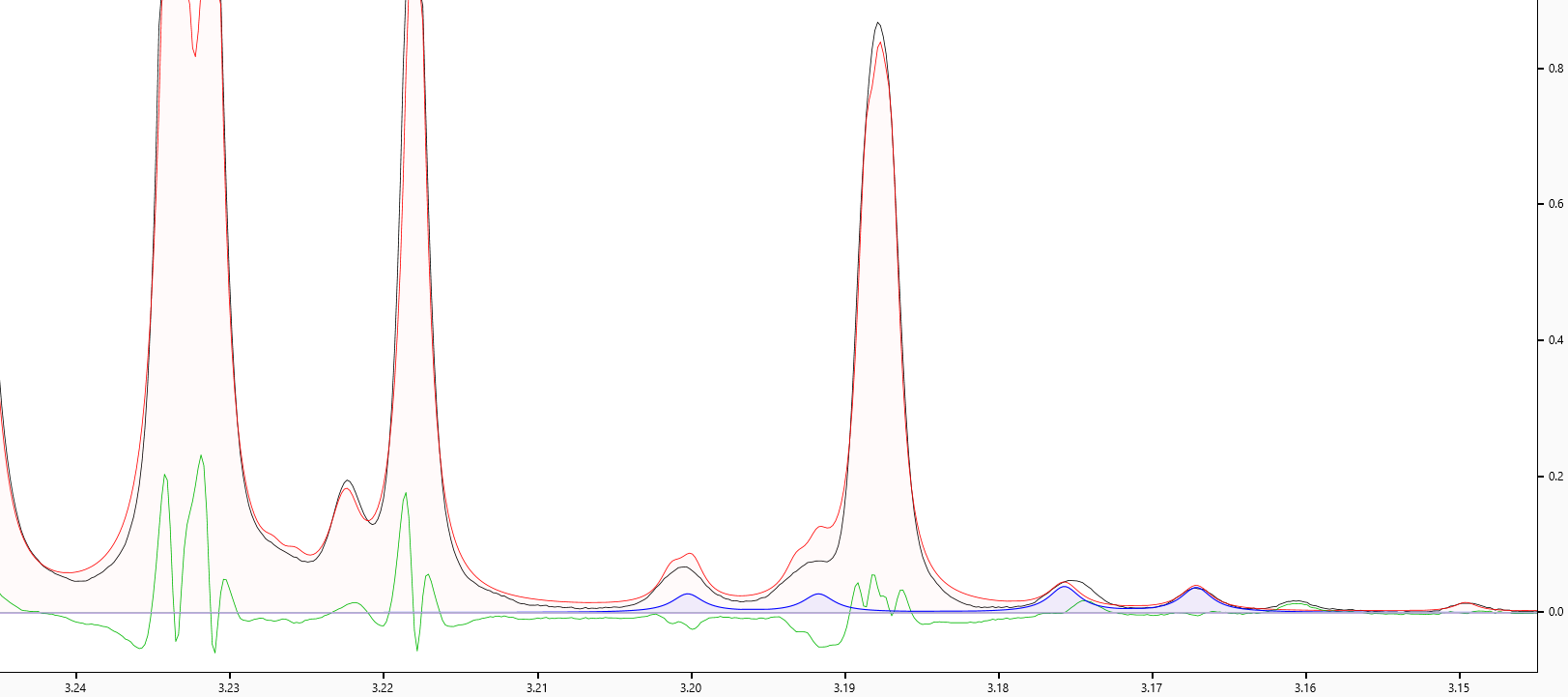

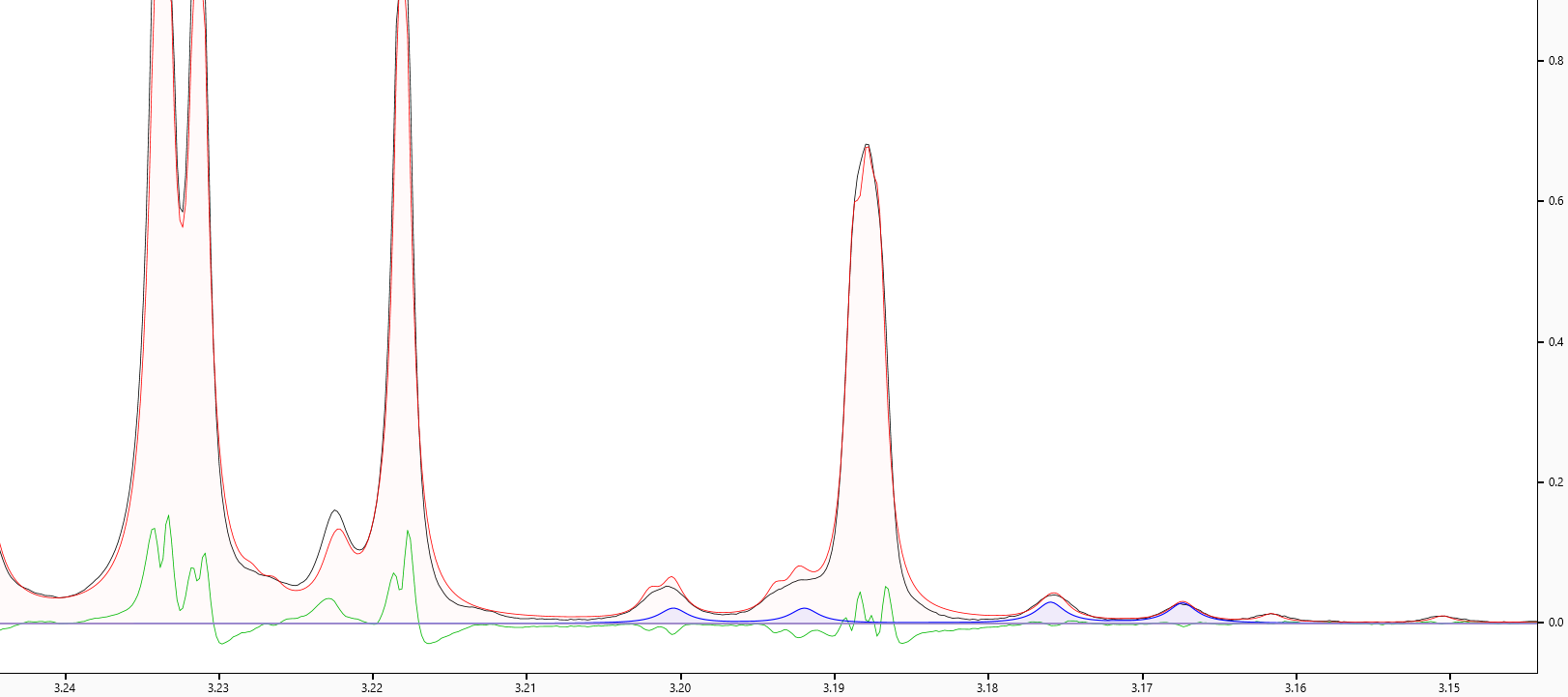


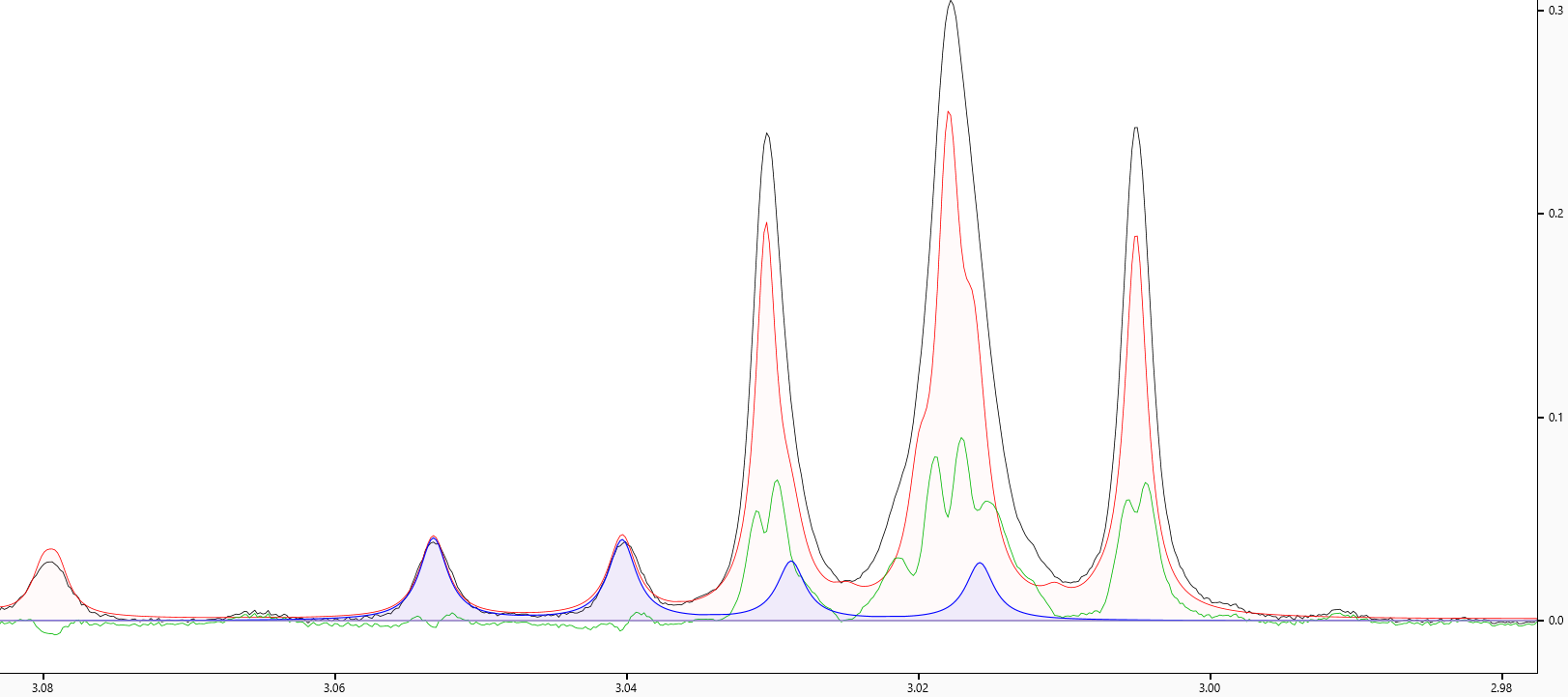

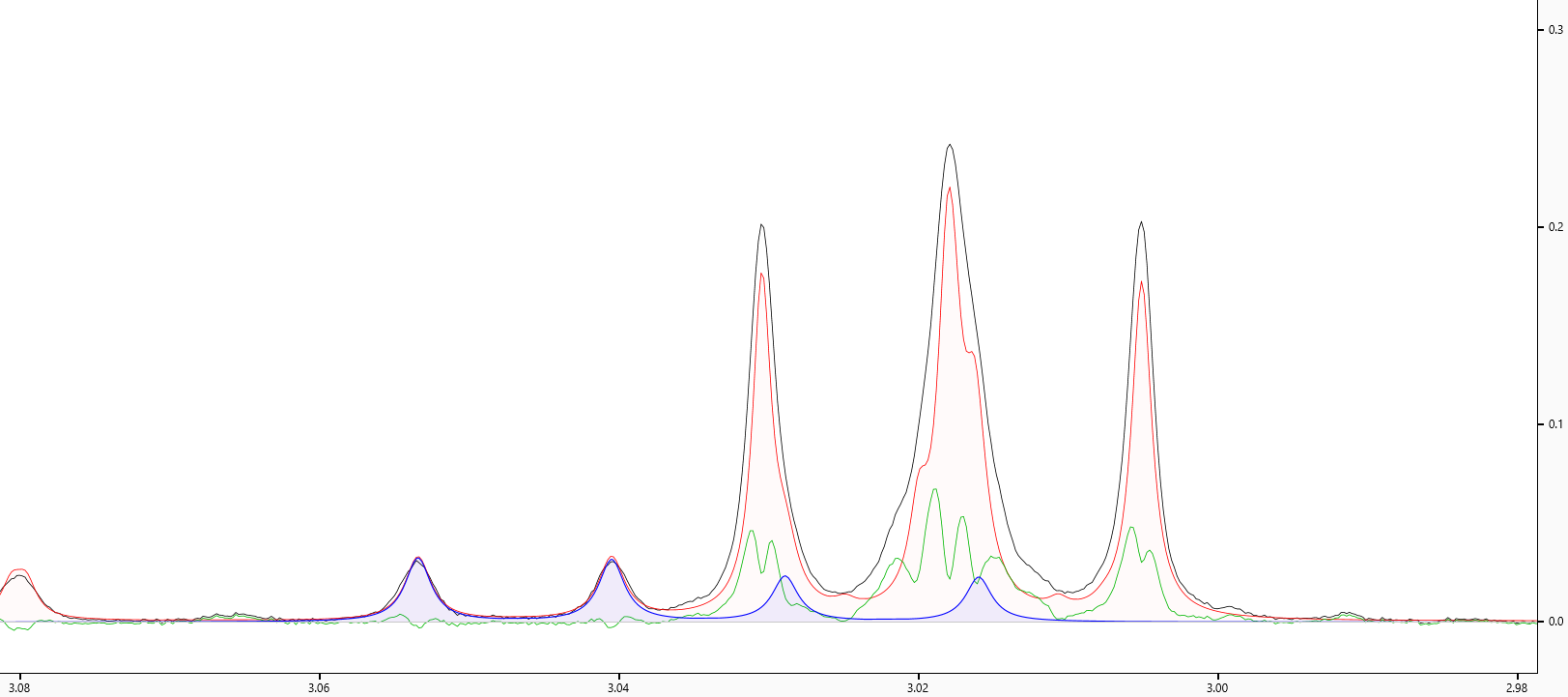

Supplement: S4 Fig — Representative profiles of the main peaks used for estimating quatification of tyrosine in O2 saturated control (left panel) and 195 mJ/cm2 irradiated (right panel) CD-CHO. (DOC) [file pone.0150957.s004.doc]
