# Supplementary material for: Impact of Dissolved Oxygen during UV-Irradiation on the Chemical Composition and Function of CHO Cell Culture Media
Source: PLoS One. 2016 Mar 14;11(3):e0150957. doi: 10.1371/journal.pone.0150957 (PMC4790850; doi:10.1371/journal.pone.0150957)
Supplement: S1 Table — Optimized MRM transitions and related instrumental parameters for the analytes monitored in this study. (DOC) [file pone.0150957.s005.doc]

| **MRM identity** | **Q1 m/z** | **Q3 m/z** | **DP (V)** | **EP (V)** | **CE(eV_lab_)** |
| --- | --- | --- | --- | --- | --- |
| Biotin | 245.0 | 227.0 | 27.0 | 10.4 | 19.2 |
| Choline | 104.0 | 60.0 | 100.0 | 10.1 | 23.0 |
| Folic acid | 442.0 | 295.0 | 100.0 | 12.0 | 20.2 |
| Lumichrome_1 | 243.0 | 172.0 | 75.0 | 9.7 | 32.9 |
| Lumichrome_2 | 243.0 | 198.0 | 75.0 | 9.7 | 33.1 |
| Niacinamide_1 | 123.0 | 80.0 | 90.0 | 9.5 | 27.2 |
| Niacinamide_2 | 123.0 | 78.0 | 90.0 | 9.5 | 30.5 |
| Niacine_2 | 124.0 | 80.0 | 100.0 | 10.2 | 31.7 |
| Niacine-1 | 124.0 | 78.0 | 100.0 | 10.2 | 27.8 |
| Pantothenic acid_1 | 220.0 | 202.0 | 45.0 | 11.2 | 15.4 |
| Pantothenic acid_2 | 220.0 | 90.0 | 45.0 | 11.2 | 18.3 |
| Pyridoxal_1 | 168.0 | 150.0 | 40.4 | 9.6 | 17.2 |
| Pyridoxal_2 | 168.0 | 122.0 | 40.4 | 9.3 | 28.1 |
| Pyridoxamine_1 | 169.0 | 152.0 | 50.0 | 9.6 | 17.3 |
| Pyridoxamine_2 | 169.0 | 134.0 | 50.0 | 9.6 | 29.2 |
| Pyridoxine_1 | 170.0 | 152.0 | 42.8 | 9.8 | 19.0 |
| Pyridoxine_2 | 170.0 | 134.0 | 42.8 | 9.4 | 29.5 |
| Riboflavin_1 | 377.0 | 243.0 | 85.0 | 10.0 | 32.6 |
| Riboflavin_2 | 377.0 | 198.0 | 85.0 | 10.0 | 49.5 |
| Taurine_1 | 126.0 | 126.1 | 50.0 | 9.6 | 7.9 |
| Taurine_2 | 126.0 | 85.0 | 50.0 | 9.6 | 13.9 |
| Thiamine_1 | 265.0 | 122.0 | 77.0 | 8.7 | 22.6 |
| Thiamine_2 | 265.0 | 144.0 | 77.0 | 8.7 | 23.2 |
| VitaminB12_1 | 678.0 | 147.0 | 93.0 | 10.0 | 41.5 |
| VitaminB12_2 | 678.0 | 359.0 | 93.0 | 10.0 | 32.8 |
