# Supplementary material for: Impact of Dissolved Oxygen during UV-Irradiation on the Chemical Composition and Function of CHO Cell Culture Media
Source: PLoS One. 2016 Mar 14;11(3):e0150957. doi: 10.1371/journal.pone.0150957 (PMC4790850; doi:10.1371/journal.pone.0150957)
Supplement: S2 Table — Additional instrumental parameters employed with the 6500 triple quadrupole mass spectrometer. (DOC) [file pone.0150957.s006.doc]

| **Instrument Parameter** | **Setting** |
| --- | --- |
| Curtain Gas | 30 psi |
| Collision Gas | 9 (arb. units) |
| Ion Spray Voltage | 5500 V |
| Source Temperature | 550°C |
| Gas 1 | 50 psi |
| Gas 2 | 50 psi |
| Interface Heater Temperature | 150°C |
| Collision Cell Exit Potential | 15 V |
