# Supplementary material for: Impact of Dissolved Oxygen during UV-Irradiation on the Chemical Composition and Function of CHO Cell Culture Media
Source: PLoS One. 2016 Mar 14;11(3):e0150957. doi: 10.1371/journal.pone.0150957 (PMC4790850; doi:10.1371/journal.pone.0150957)
Supplement: S3 Table — (DOC) [file pone.0150957.s007.doc]

| **Vitamin** | **Retention Time (min)** |
| --- | --- |
| Choline | 0.69 |
| Niacinamide | 2.57 |
| Niacin | 1.43 |
| Pyridoxal | 1.69 |
| Pyridoxamine | 0.86 |
| Pyridoxine | 2.36 |
| Lumichrome | 9.63 |
| Biotin | 9.56 |
| Thiamine | 1.02 |
| Riboflavin | 9.59 |
| Vitamin B12 | 9.53 |
