# Supplementary material for: Impact of Dissolved Oxygen during UV-Irradiation on the Chemical Composition and Function of CHO Cell Culture Media
Source: PLoS One. 2016 Mar 14;11(3):e0150957. doi: 10.1371/journal.pone.0150957 (PMC4790850; doi:10.1371/journal.pone.0150957)
Supplement: Supplementary file 5 [file pone.0150957.s002.doc]

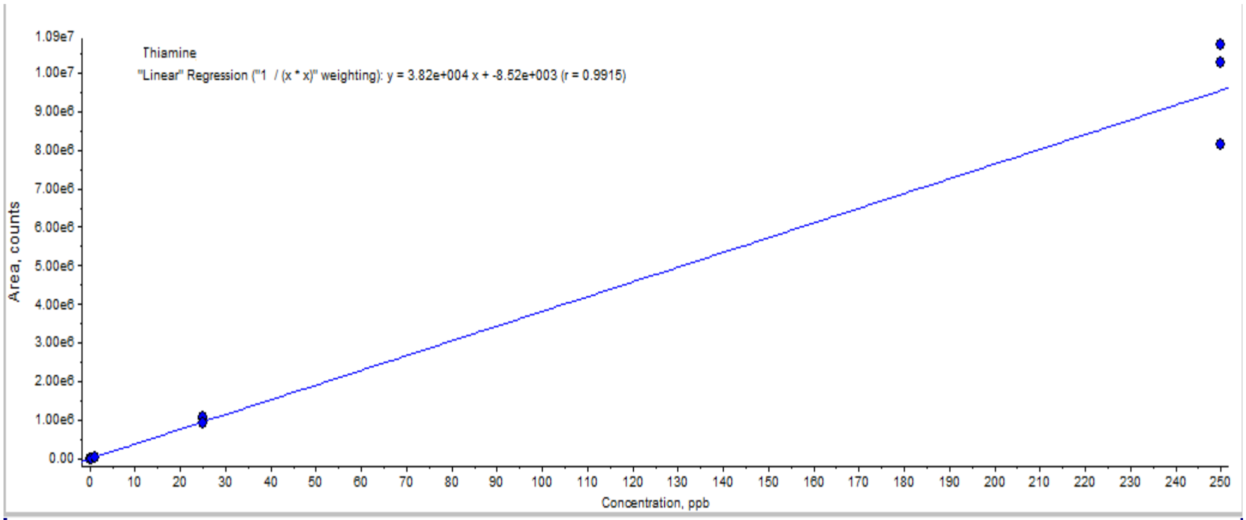
Thiamine
"Linear" Regression ("1 / (x * x)" weighting): y = 3.82e+004 x + -8.52e+003 (r = 0.9915)
Area, counts
Concentration, ppb

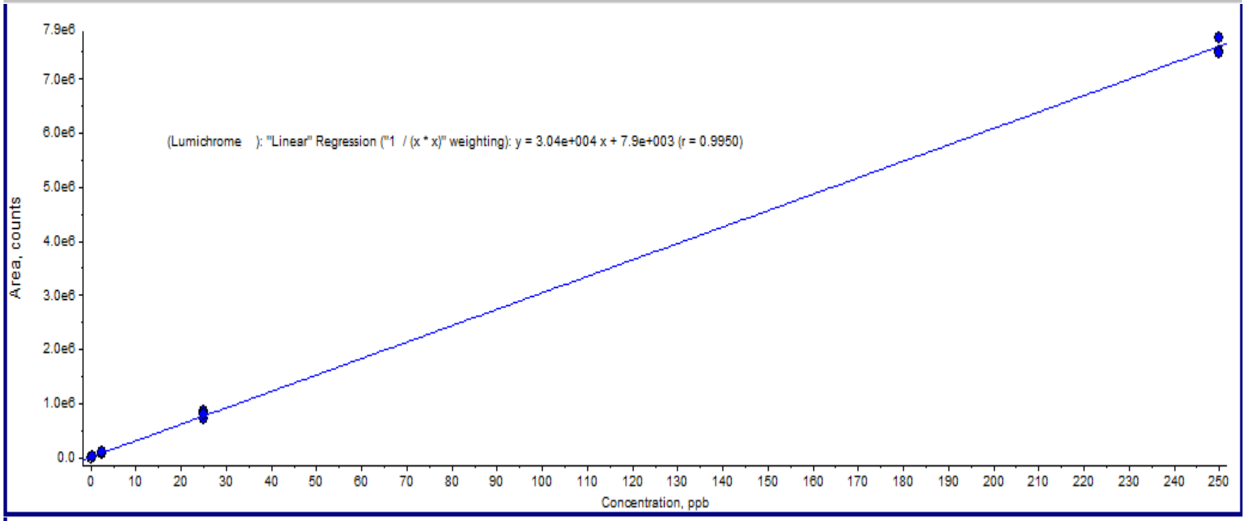
(Lumichrome): "Linear" Regression ("1 / (x * x)" weighting): y = 3.04e+004 x + 7.9e+003 (r = 0.9950)
Area, counts
Concentration, ppb

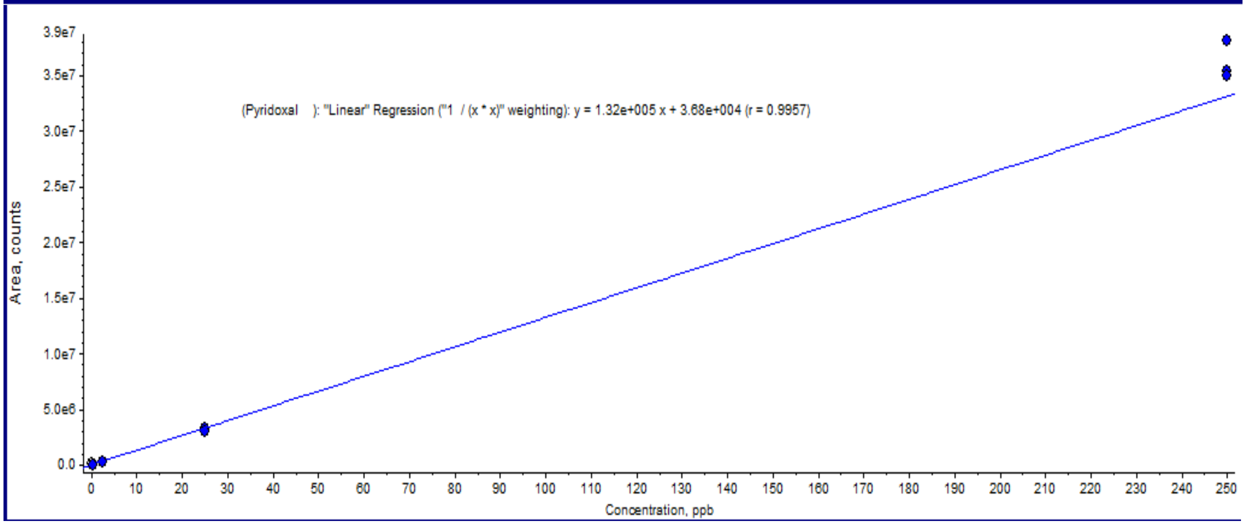
(Pyridoxal): "Linear" Regression ("1 / (x * x)" weighting): y = 1.32e+005 x + 3.68e+004 (r = 0.9957)
Area, counts
Concentration, ppb

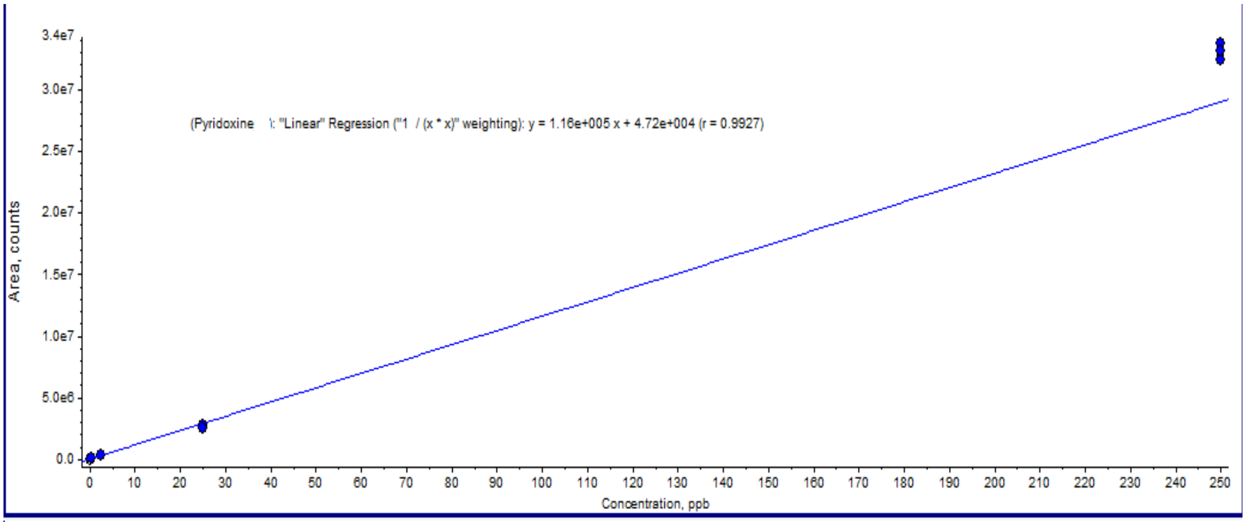
(Pyridoxine): "Linear" Regression ("1 / (x * x)" weighting): y = 1.16e+005 x + 4.72e+004 (r = 0.9927)
Area, counts
Concentration, ppb

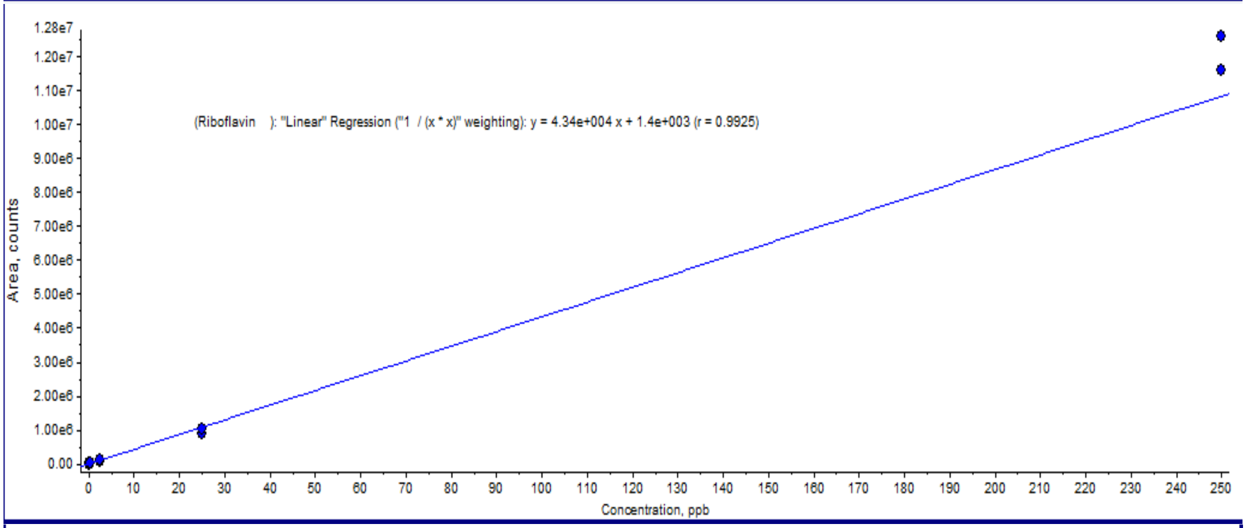
(Riboflavin): "Linear" Regression ("1 / (x * x)" weighting): y = 4.34e+004 x + 1.4e+003 (r = 0.9925)
Area, counts
Concentration, ppb

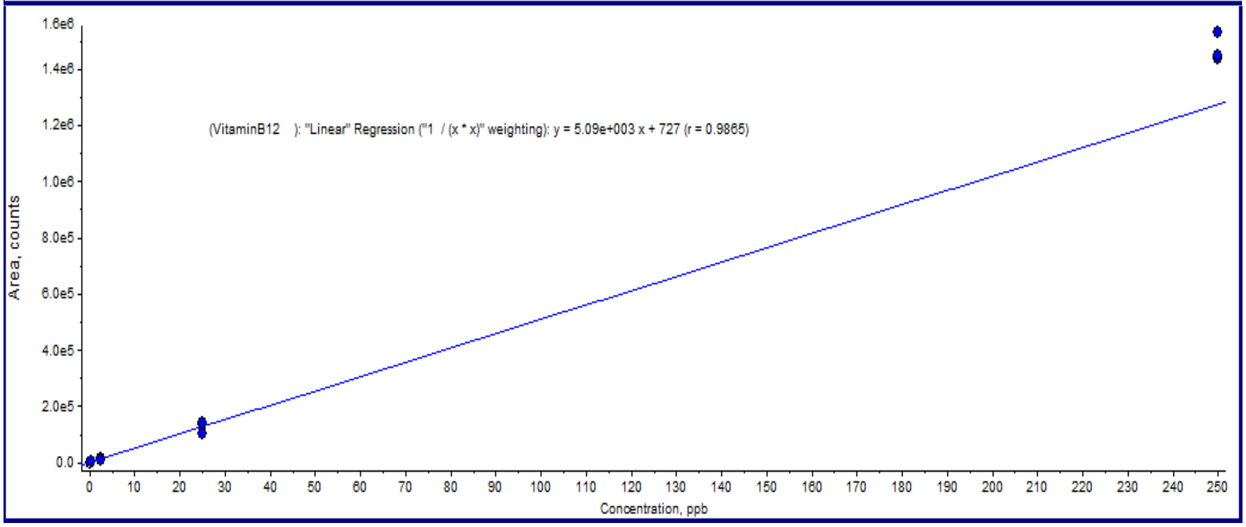
(VitaminB12): "Linear" Regression ("1 / (x * x)" weighting): y = 5.09e+003 x + 727 (r = 0.9865)
Area, counts
Concentration, ppb

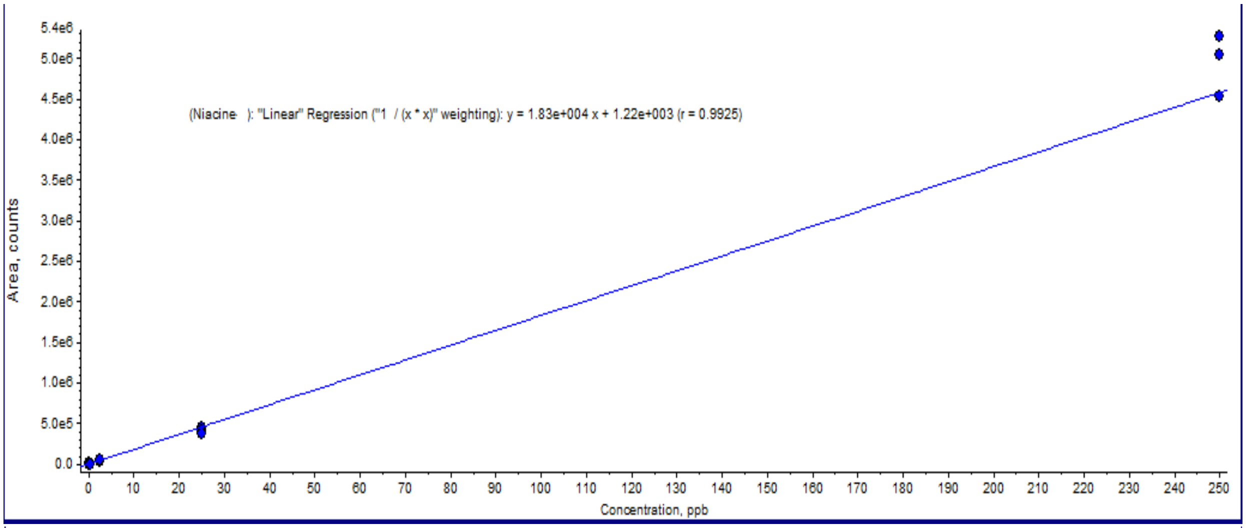
(Niacine): "Linear" Regression ("1 / (x * x)" weighting): y = 1.83e+004 x + 1.22e+003 (r = 0.9925)
Area, counts
Concentration, ppb

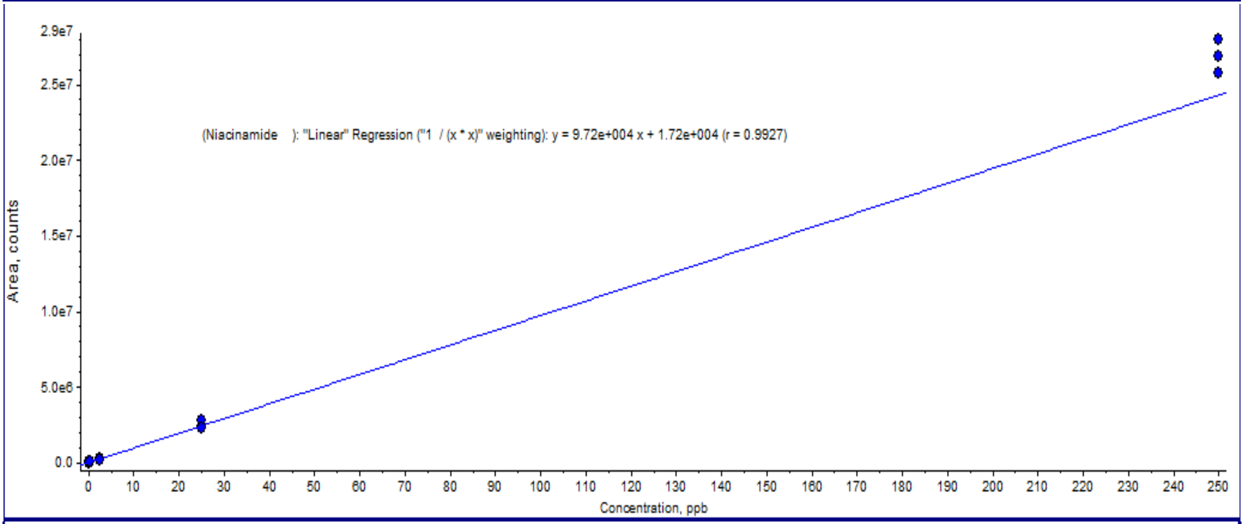
(Niacinamide): "Linear" Regression ("1 / (x * x)" weighting): y = 9.72e+004 x + 1.72e+004 (r = 0.9927)
Area, counts
Concentration, ppb

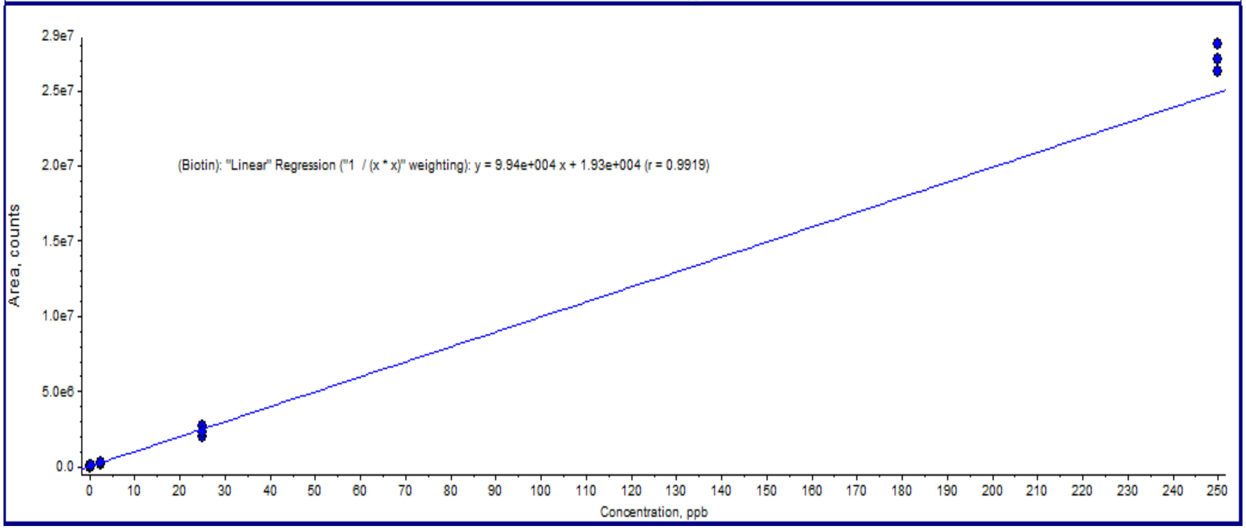
(Biotin): "Linear" Regression ("1 / (x * x)" weighting): y = 9.94e+004 x + 1.93e+004 (r = 0.9919)
Area, counts
Concentration, ppb

1
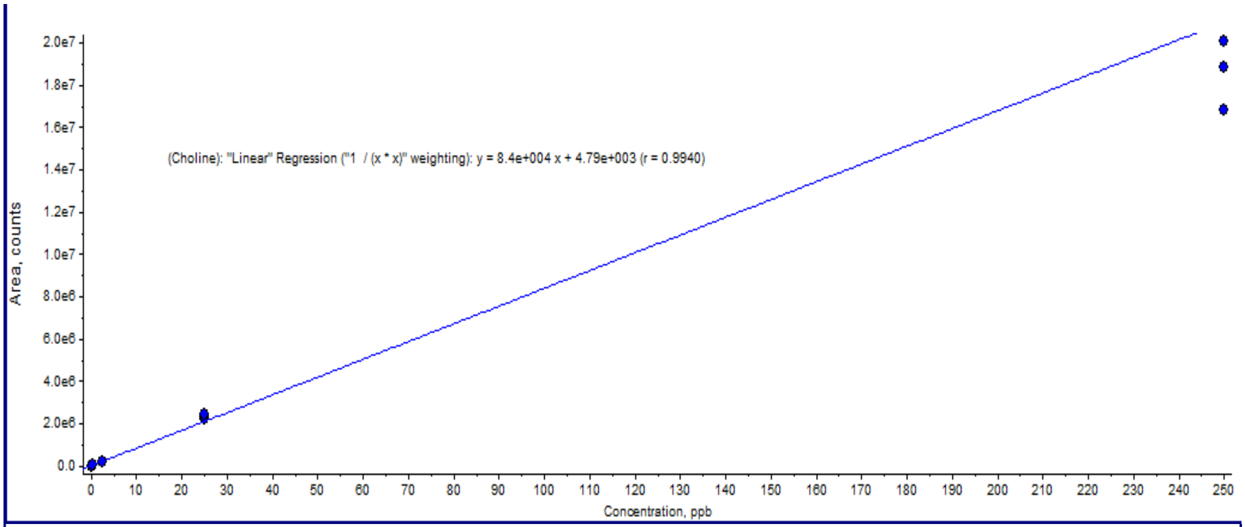
(Choline): "Linear" Regression ("1 / (x * x)" weighting): y = 8.4e+004 x + 4.79e+003 (r = 0.9940)
Area, counts
Concentration, ppb

2
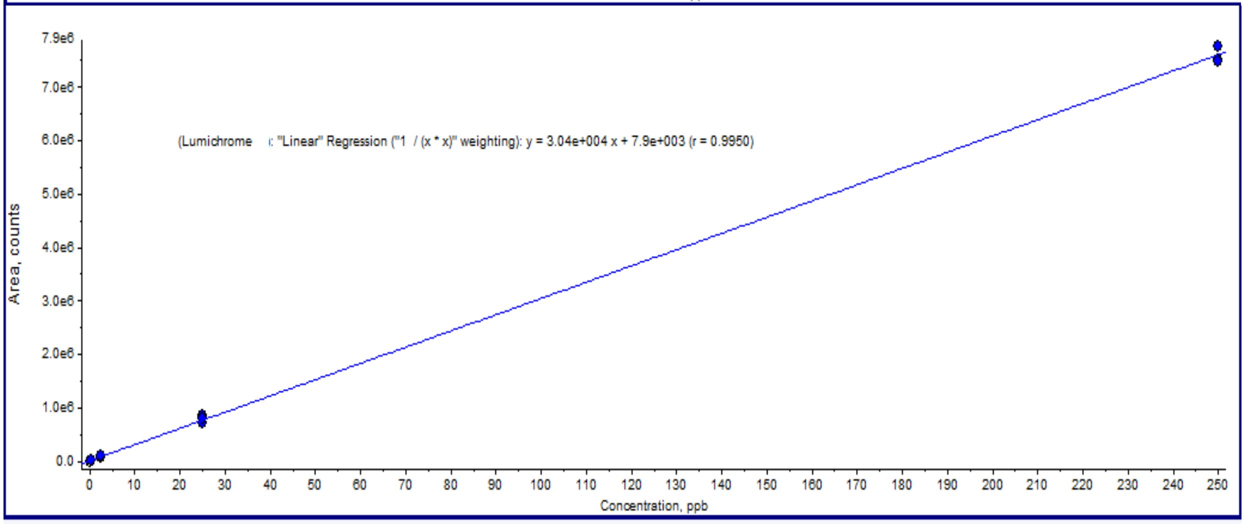
(Lumichrome : "Linear" Regression ("1 / (x * x)" weighting): y = 3.04e+004 x + 7.9e+003 (r = 0.9950)
Area, counts
Concentration, ppb

1
2
